# Identification of vacuoles containing extraintestinal differentiated forms of *Legionella pneumophila* in colonized *Caenorhabditis elegans* soil nematodes

**DOI:** 10.1002/mbo3.271

**Published:** 2015-07-01

**Authors:** Jacqueline R Hellinga, Rafael A Garduño, Jay D Kormish, Jennifer R Tanner, Deirdre Khan, Kristyn Buchko, Celine Jimenez, Mathieu M Pinette, Ann Karen C Brassinga

**Affiliations:** 1 Department of Microbiology, Faculty of Science, University of ManitobaWinnipeg, Manitoba, Canada, R3T 2N2; 2 Department of Microbiology and Immunology, Faculty of Medicine, Dalhousie UniversityHalifax, Nova Scotia, Canada, B3H 1X5; 3 Department of Medicine, Faculty of Medicine, Dalhousie UniversityHalifax, Nova Scotia, Canada, B3H 1X5; 4 Department of Biological Sciences, Faculty of Science, University of ManitobaWinnipeg, Manitoba, Canada, R3T 2N2

**Keywords:** Cyst biogenesis, LCV, *Legionella*, nematode, oocyte endocytosis pathway, protozoa

## Abstract

*Legionella pneumophila*, a causative agent of Legionnaires’ disease, is a facultative intracellular parasite of freshwater protozoa. *Legionella pneumophila* features a unique developmental network that involves several developmental forms including the infectious cyst forms. Reservoirs of *L. pneumophila* include natural and man-made freshwater systems; however, recent studies have shown that isolates of *L. pneumophila* can also be obtained directly from garden potting soil suggesting the presence of an additional reservoir. A previous study employing the metazoan *Caenorhabditis elegans*, a member of the *Rhabditidae* family of free-living soil nematodes, demonstrated that the intestinal lumen can be colonized with *L. pneumophila*. While both replicative forms and differentiated forms were observed in *C. elegans*, these morphologically distinct forms were initially observed to be restricted to the intestinal lumen. Using live DIC imaging coupled with focused transmission electron microscopy analyses, we report here that *L. pneumophila* is able to invade and establish *Legionella*-containing vacuoles (LCVs) in the intestinal cells. In addition, LCVs containing replicative and differentiated cyst forms were observed in the pseudocoelomic cavity and gonadal tissue of nematodes colonized with *L. pneumophila*. Furthermore, establishment of LCVs in the gonadal tissue was Dot/Icm dependent and required the presence of the endocytic factor RME-1 to gain access to maturing oocytes. Our findings are novel as this is the first report, to our knowledge, of extraintestinal LCVs containing *L. pneumophila* cyst forms in *C. elegans* tissues, highlighting the potential of soil-dwelling nematodes as an alternate environmental reservoir for *L. pneumophila*.

## Introduction

The gram-negative bacterium *Legionella pneumophila* is responsible for the majority of Legionnaires’ disease cases reported worldwide (Fields et al. 2002; Newton et al. 2010). As a facultative intracellular parasite of freshwater protozoa, *L. pneumophila* possesses a unique developmental cycle that in its simplest dimorphic conception alternates between vegetative replicative forms and resilient motile cyst forms (or MIFs, for mature infectious forms) (Berk et al. 1998; Garduño et al. 2002). However, it is now accepted that *L. pneumophila* differentiates along a developmental network that is far more complex than a simple dimorphic developmental cycle (Garduño 2008; Robertson et al. 2014). Cyst biogenesis occurs postexponentially and is coordinated with the expression of virulence traits that include increased infectivity, cytotoxicity, and osmotic resistance (Molofsky and Swanson 2004; Newton et al. 2010). The precise number and identity of the signals that trigger formation of cyst forms are not currently known; however, it appears that the onset of nutrient-limited conditions is a key factor contributing to cyst biogenesis (Byrne and Swanson 1998; Edwards et al. 2009; Hovel-Miner et al. 2010; Fonseca and Swanson 2014). Shortly after release from the host cell, the cyst form drops its polar flagellum and becomes metabolically dormant. The cyst forms are highly resistant to the effects of detergents and antibiotics, and are hyper-infectious as shown by cell-based infection models (Garduño et al. 2002). Ultrastructural analyses *via* transmission electron microscopy (TEM) of the several developmental forms produced in HeLa cells revealed the replicative forms as typical Gram-negative rods, whereas the irregularly shaped coccoid cyst forms featured unique characteristics that included thickened cell walls, multiple membrane laminations and large cytoplasmic inclusions of poly-*β*-hydroxybutyrate (PHBA) (Faulkner and Garduño 2002). Full development of the multiphasic cycle as described in HeLa cells has also been demonstrated in protozoa (Cirillo et al. 1994; Greub and Raoult 2003), but not in lymphoid human cell lines including U937 and THP-1 (Abdelhady and Garduño 2013). In broth culture, the replicative forms only transition into stationary phase forms once nutrients have been spent, indicating that the signals that trigger cyst biogenesis are protozoan- or HeLa cell specific (Garduño et al. 2002; Fonseca and Swanson 2014). In comparison to cyst forms, in vitro grown stationary phase forms are up to 1000-fold less infectious to HeLa cells and macrophages, and possess few PHBA inclusion bodies (Cirillo et al. 1999; Garduño et al. 2002). Thus, it is proposed that free cyst forms, when aerosolized in water droplets and inadvertently inhaled by a susceptible individual, infect alveolar macrophages causing Legionnaires’ disease.

Reservoirs of *L. pneumophila* normally include natural and anthropogenic freshwater systems where protozoa thrive; however, isolates of *L. pneumophila* have been obtained directly from garden potting soil suggesting the presence of additional reservoirs (den Boer et al. 2007; Casati et al. 2009; Brassinga et al. 2010). Free-living soil nematodes of the *Rhabditidae* family, for instance the well-studied *Caenorhabditis elegans*, are normal habitants of compost-rich soil; they are bacterivorous and migrate throughout the soil matrix detecting food using chemosensation (Grewal and Wright 1992; Young et al. 1996, 1998; Rodger et al. 2004). The pharynx of *C. elegans* is a muscular pumping structure that functions to bring bacteria into the digestive tract of the nematode (Albertson and Thomson 1976). Although the pharyngeal grinders disrupt most of the ingested bacteria, a portion of the bacteria can survive passage into the intestinal lumen. Bacteria are either eliminated immediately from the intestinal lumen by rhythmic peristaltic defecation dispersing viable bacteria into the soil or establish colonization providing a steady source of bacteria to be eliminated by defecation (Avery 1993; Anderson et al. 2006; Fang-Yen et al. 2009). While a few studies have reported on the potential role of bacterivorous soil nematodes in harboring and disseminating viable pathogenic bacteria into the soil, this ecological niche warrants further investigation (Gibbs et al. 2005; Anderson et al. 2006). A recent report employing the soil nematode *C. elegans* demonstrated that the intestinal lumen can be colonized with *L. pneumophila* in a moist simulated soil environment supporting the notion that soil nematodes can serve as environmental reservoirs of *L. pneumophila* (Brassinga et al. 2010). TEM analyses indicated that the bacteria underwent morphologic differentiation that included some, but not all, of the ultrastructural features similar to cyst forms observed in the protozoan or HeLa cell model (Faulkner and Garduño 2002; Brassinga et al. 2010). However, bacterial replication and morphological differentiation were only observed to occur extracellularly in the intestinal lumen as no invading bacteria were found in the surrounding tissue. This finding was contradictory to the intracellular lifestyle of *L. pneumophila* in protozoa; however, it was presumed that the requisite nutrients were available in the intestinal lumen facilitating growth and morphological differentiation (Brassinga et al. 2010).

Here, we report that in nematodes colonized with *L. pneumophila*, the bacteria are able to invade and establish *Legionella*-containing vacuoles (LCVs) in intestinal cells. Not only is invasion of the intestinal cells documented, but LCVs containing replicative forms are also observed in the pseudocoelomic cavity early in the colonization process suggesting a means for dissemination of LCVs throughout the body cavity of the nematode. LCVs containing fully differentiated cyst forms retained in vacuoles are highly similar to those formed in HeLa cells and protozoa infected with *L. pneumophila* as reported elsewhere (Faulkner and Garduño 2002; Greub and Raoult 2003). Uptake and establishment of LCVs into a secondary infection site, the gonadal tissue, is dependent on the endocytic factor RME-1 and the Dot/Icm system, respectively. Taken together, our findings are novel as this is the first report of the ability of *L. pneumophila* to morphologically differentiate within the extraintestinal tissues of *C. elegans* highlighting the potential of soil-dwelling nematodes, such as *C. elegans*, as an alternate environmental reservoir for *L. pneumophila*.

## Experimental Procedures

### Bacterial and nematode strains, media, and general methods

Bacterial strains used in this study are listed in Table1. *Legionella pneumophila* Lp02 and derivative strains were cultured on buffered charcoal yeast extract (BCYE) agar supplemented with thymidine (100 *μ*g/mL), and streptomycin (100 *μ*g/mL) where appropriate, from frozen stock (BYE broth containing 10% dimethyl sulfoxide) and incubated at 37°C with 5% CO_2_ for 3–4 days (Feeley et al. 1979; Berger and Isberg 1993). *Escherichia coli* host strains DH5*α* and DH5*αλ*pir strains used for cloning and allelic gene exchange strategies, respectively, were grown in Lysogeny broth (LB) media (broth or agar) at 37°C. Kanamycin (40 *μ*g/mL) and ampicillin (100 *μ*g/mL) were added to LB media (broth or agar) where appropriate. *Caenorhabditis elegans* strains were maintained on nematode growth media (NGM), supplemented with *E. coli* OP50 as a food source, at 15°C using standard manipulation methods (Hope 1999).

**Table 1:** List of *Caenorhabditis elegans* and bacterial strains, and plasmids used in this study

Strain	Description	Reference or source
*Escherichia coli*		
DH5*α*	F’ *endA1 hsdR17*(*r*_*k*_*- m*_*k*_*-*) *supE44 thi-1 recA1 gyrA* (*Nal*^*r*^) *relA1 Δ*(*lacZYA-argF*)*U169 deoR*(ɸ80lacΔ(lacZ)M15)	New England Biolabs
DH5*αλ*pir	K-12 F- *φ*80*lacZ*ΔM15 *endA recAhsdR17* (*rm- mK+*)*supE44 thi-1 gyrA96 relA1 Δ*(*lacZYA-argF*) U169 *λpir*	M. Swanson (Bryan et al. 2013)
OP50	Uracil auxotroph	CGC (Brenner 1974)
HT115(DE3)	rnc14::ΔTn10*λ*(DE3)	J. Kinchen (Timmons et al. 2001)
MP375	HT115 pL4440::*ced-3*, Amp^r^ Tet^r^	This study
*unc-22* RNAi	HT115 pL4440::*rme-2*, Amp^r^ Tet^r^	Arhinger RNAi library (ZK617.1 [F03 IV])
*Legionella pneumophila*		
Lp02	Str^r^, Thy^−^, HsdR^−^ derivative of Philadelphia-1 strain	M. Swanson (Berger and Isberg 1993)
Lp03	Lp02 *dotA03 thyA*^−^	R. Isberg (Berger and Isberg 1993)
Lp03 comp	Lp03 pKB9	R. Isberg (Roy et al. 1998)
Lp02 Δ*sdhA*	Deletion of *sdhA*	R. Isberg (Laguna et al. 2006)
Lp02 Δ*sdhA* comp	Lp02 pRL101	R. Isberg (Laguna et al. 2006)
KB421	Lp02 Δ*magA*::*gfpmut3*	This study
Lp02 *thyA* comp	Lp02 pBH6119	
Plasmids		
pSR47s	oriTRP4 oriR6K kan sacB suicide vector	J. Vogel (Merriam et al. 1997)
pSK	Cloning vector, Amp^r^	Stratagene
pKB127	pBH6119::P_*magA*_ 245-bp region cloned into BamHI and XbaI sites, Amp^r^	Morash et al. (2009)
pDK268	pBH6119::P_*magA*_ 807-bp region cloned into BamHI and XbaI sites, Amp^r^	This study
pDK289	pSK::P_*magA*_ 807-bp region and *gfpmut3* cloned into BamHI and SacII sites, Amp^r^	This study
pDK298	Downstream 817-bp region flanking *magA* cloned into SacII and SacI sites of pDK289, Amp^r^	This study
pKB398	pSR47s:: P_*magA*_ 807-bp region, *gfpmut3* and downstream flanking 817-BP region cloned into SalI and SacI sites; Kan^r^	This study
pKB9	*dotA* expression plasmid; Amp^r^	R. Isberg (Roy et al. 1998)
pRL101	*sdhA* expression plasmid; Amp^r^	R. Isberg (Laguna et al. 2006)
pL4440	RNAi cloning vector for generating dsRNA; Amp^r^, Tet^r^	J. Kinchen (Timmons et al. 2001)
pMP371	pL4440::*ced-3*	This study
pBH6119	Promoterless GFP vector; Amp^R^, Thy^+^	M. Swanson (Hammer and Swanson 1999)
*C. elegans*		
N2	Wild-type isolate from Bristol, England	CGC (Brenner 1974)
GH403	*glo-3*(*kx94*)	CGC (Rabbitts et al. 2008)
DH1201	*rme-1*(*b1045*)	CGC (Grant et al. 2001)
MD701	*P*_*lim-7*_*ced-1::gfp*	CGC (Schumacher et al. 2005)

### Molecular methods, reagents and sequencing

Oligonucleotides, synthesized by Invitrogen (Life technologies, Grand Island, NY), are listed in Table2. All restriction and modifying enzymes, dNTPs, Taq Polymerase, Phusion Taq polymerase and Q5 High Fidelity Polymerase were purchased from New England Biolabs (Whitby, Ontario, Canada). Chemical reagents were procured from Sigma Aldrich (St. Louis, MO), Fisher Scientific (Ottawa, Ontario, Canada), and VWR (Mississauga, Ontario, Canada). Plasmid DNA and DNA gel extraction were isolated using QIAprep Spin Miniprep and Gel Extraction kits, respectively, from Qiagen (Toronto, Ontario, Canada). General cloning protocols were used to construct and manipulate plasmid vectors (Sambrook 2001). For sequencing, samples were sent to The Centre for Applied Genomics (TCAG) at the Hospital for Sick Children (Toronto, Ontario).

**Table 2:** Oligonucleotides used in this study

Purpose	Primers (5′→3′ direction)^1^	Annealing temp. (°C)	Length (bp)
Amplicon			
P_magA_	PF GCGATAggatccGTAGCTGATTAATTGA	51.5	807
	PR GCGATAtctagaGTAGCTGATTAATTGA		
P_magA_ + gfpmut3	P_magA_ PF	57.5	1362
	PR GCGATAccgcggTTATTTGTACAATTCA		
3′ magA flank	PF GCGATAccgcggATTATTCTAAGGGGTG	52.1	817
	PR GCGATAgagctcCGTCAGTATAGCCTT A		
P_magA_ + gfpmut3 + 3′magA flank	PF GCGATAgtcgacGTAGCTGATTAATTGA	57.5	2179
	3′ magA flank PR		
magA Int	PF GCACTGGAAGCCTACA	57.5	273
	PR GATGTCGAACTCACTCA		
ced-3	PF CATggcgcgccATGAGCGATTACGACTTGAACTGTA	51.5	595
	PR CATactagtCGTCAAGATAGAAGGAGCTTGCTA		

### Construction of chromosomally expressed GFP-tagged *L. pneumophila* strain

Chromosomal replacement of *magA* with *gfpmut3* was achieved as follows. Plasmid construct pKB127 was digested with BamHI and XbaI to remove the 245-bp P_*magA*_ region and religated with the 807-bp P_*magA*_ amplicon amplified from Lp02 genomic DNA creating pDK268. The 807-bp P_*magA*_ promoter region along with *gfpmut3* was amplified by polymerase chain reaction (PCR) from pDK268 generating the P_*magA*_* *+ *gfpmut3* amplicon for cloning into BamHI and SacII sites on pSK creating pDK289. The downstream region flanking the 3′ side of *magA* was amplified by PCR from Lp02 genomic DNA and the resultant 3′ *magA* flank amplicon was cloned into SacII and SacI sites on pDK289 creating pDK298. The P_*magA*_ + *gfpmut3 *+* *3′*magA* flank amplicon generated by PCR from pDK298 was then cloned into Sal and SacI sites on pSR47s generating the allelic suicide vector construct pKB398 for electroporation into Lp02 and plating on BCYE supplemented with thymidine and kanamycin to select for the first cross-over event. To select for the second cross-over event, transformant colonies were patched on to BCYE supplemented with thymidine and sucrose. Colonies that grew in the presence of sucrose were then replica plated onto BCYE + thymidine and BCYE + thymidine + kanaymcin, respectively, and colonies that were kanamycin sensitive (Kan^S^) were selected for further screening by PCR for the absence of *magA* using the *magA* (int) primer set (Table2). Positive Δ*magA::gfpmut3* colonies were subjected to DNA sequencing to verify deletion of *magA* and epifluorescence microscopy to verify GFP expression.

### RNAi-treated nematodes

NGM plates supplemented with 12.5 *μ*g/mL carbenicillin and 8 *μ*mol/L Isopropyl beta-D-1-thiogalactopyranoside (IPTG) were spotted with 50 *μ*L of overnight cultures of MP375 (*ced-3* RNAi) or *unc-22* RNAi (control) strains and incubated at 37°C overnight. When cooled to room temperature, the plates were seeded with 2–3 gravid *ced-1::gfp* (MD701) nematodes. After overnight egglay at 20°C, the adult nematodes were removed and eggs allowed to hatch and molt to L4 stage for seeding of survival assay plates. Successful induction of dsRNA of *ced-3* was correlated with visual observation of the “shaking” phenotype of *unc-22* RNAi-treated nematodes.

### Survival assay

Survival assays were conducted in accordance to the protocol reported in Brassinga and Sifri (2013). Complemented strains were grown on BCYE media lacking thymidine. Briefly, ∼30 nematodes were seeded at larval stage L4 onto triplicate BCYE assay plates containing centralized 48-h grown bacteria lawns 15 mm in diameter (i.e., spotted) and monitored on a daily basis for survival for up to 14 days. Nematodes were transferred onto fresh spotted assay plates to separate subjects from progeny. Nematodes were considered dead if their pharyngeal pumping ceased and they were not responsive to touch with a platinum pick. Nematodes that crawled off the plate were not included in the analysis. Nematode survival was calculated by the Kaplan–Meier method and differences were tested for significance using the log-rank test (GraphPad Software, San Diego, CA, USA.).

### Live microscopy

Nematodes were seeded per the survival assay protocol. Over a 9-day period after initial seeding, ∼20 nematodes were collected at 24 h intervals, and washed in M9 buffer (22 mmol/L KH_2_PO_4_, 42 mmol/L Na_2_HPO_4_, 86 mmol/L NaCl, 1 mmol/L MgSO_4_) in a sterile 60 × 15 mm petri plate. Nematodes were anesthetized in10 mmol/L levamisole (in M9 buffer) on a 2% agarose pad mounted on a glass microscope slide after which a glass coverslip was placed and sealed with silicon grease. Nematodes and protozoa were imaged with 40X/1.4 and 63X/1.4 oil objectives and a 150X/1.35 glycerin objective, respectively, on a Zeiss Observer Z1 Inverted Microscope (Jena, Germany) equipped with Normaski optics and epifluorescence, and operated using AxioVision Rel 4.8 or Zen software. Sill image and video files were saved in *.zvi format and converted into *.jpeg or *.avi formats, respectively. Corpse count assays were conducted as reported in Brassinga et al. (2010) with the exception that *ced-1*::GFP nematodes (MD701) were employed with enumeration done by fluorescence microscopy.

### Transmission electron microscopy

Nematodes were seeded as per the survival assay protocol. Six days after the initial seeding, ∼30–40 nematodes were collected, washed in M9 buffer and fixed in accordance to the established conventional two-step fixation protocol (Hall 1995). Briefly, the samples were primarily fixed with Modified Karnovsky Fixative overnight at 4°C after which a postfix of 1% Osmium tetroxide for 1 h was applied followed by a distilled water rinse. Nematodes were placed in a side-by-side fashion in the same orientation (anterior to posterior) relative to each other, and embedded with 3% molten agarose. Agarose blocks were subjected to dehydration in an ethanol (50-75-95-100%) series with a final dehydration in 100% propylene oxide. The blocks were embedded in Embed 812 resin for 48 h with fresh resin changes every 2–5 h after which the samples were placed in fresh resin in embedding molds, allowed to harden and removed for curing 60°C for 3 days. Ultrathin sections (cross and longitudinal) ∼70 nm in thickness, obtained with a diamond knife, were collected on 300 mesh copper grids. The grids were examined in a Hitachi TM-1000 Electron Microscope (Schaumburg, IL, USA). Images were acquired using an Advanced Microscopy Techniques (AMT, Woburn, MA, USA) camera model 1600 mol/L with software AMT Image Capture Engine V601.

### Immunogold staining

Nematodes were seeded as per the survival assay protocol. Six days after the initial seeding, nematodes were collected and washed in M9 buffer. Two sample fixation treatments were considered: formaldehyde and osmium. Osmium is better for image contrast; however, there was concern that the antibody would not interact with osmium-fixed antigens and thus, fixation with formaldehyde was implemented, which typically does not affect antibody interactions. In our hands, we found both fixation treatments to be comparable with respect to the labeling and image quality.

For the formaldehyde treatment, approximately half of the nematode samples were primarily fixed with 4% formaldehyde-0.25% glutaraldehyde fixative in 0.1 mol/L cacodylate buffer for 1 h then washed with distilled water. For the osmium treatment, the second half of nematode samples were fixed in 2.5% Glutaraldehyde in 0.1 mol/L sodium cacodylate buffer for 1 h, washed with distilled water, then washed three times (10 min each) in 0.5% OsO4, 0.5% KFe(CN)6 in 0.1 mol/L cacodylate buffer, and then washed with distilled water. For both sample treatments, nematodes were placed in a side-by-side fashion in the same orientation (anterior to posterior) relative to each other on a solidified 3% agarose surface, and embedded with 3% molten agarose as established in two-step conventional fixation (Hall 1995). Agarose blocks were subjected to a dehydration ethanol (50-75-95-100%) series. The samples in 95% ethanol were processed based on the protocol outlined in Faulkner and Garduño (2013). For both sample treatments, ultrathin sections of 70 nm in thickness were obtained with a diamond knife and collected on 150 mesh nickel grids.

Tagging of EM sections with an Lp1-specific primary rabbit antibody and a gold-conjugated anti-rabbit secondary donkey antibody was done in accordance to established protocols (Faulkner and Garduño 2013). The grids were examined in a JEOL JEM-1230 TEM equipped with a Hamamatsu (Middlesex, NJ, USA) ORCA-HR high-resolution (2000 by 2000 pixels) digital camera, or a Hitachi TM-1000 Electron Microscope, equipped with an AMT CCD camera model 1600 mol/L with software AMT Image Capture Engine V601.

### Protozoa maintenance and infection with *L. pneumophila*

*Acanthamoeba castellanii* ATCC 30234 was maintained in proteose peptone yeast-extract glucose (PYG) broth (pH 6.5) in a T75 cell culture flask with a vented filter cap at 25°C. Lp02 *thyA* comp strain was utilized as the plasmid borne *thyA* (encoding thymidylate synthase) provides the necessary thymidine to rescue the auxotrophic phenotype of Lp02 strain. When confluent, protozoan cells were washed twice with 10 mL of fresh Ac buffer (PYG broth lacking proteose peptone, yeast-extract and glucose) (Moffat and Tompkins 1992) and resuspended at a concentration of 1 × 10^5^–1 × 10^6^ cells/mL in fresh Ac buffer in a 24 well plate. Following a 1-h incubation at 25°C to allow adherence of protozoa to the well bottoms, the protozoa was infected with Lp02 *thyA* comp at a multiplicity of infection (MOI) of 0.1 for 1 h at 25°C after which the cells were washed twice with Ac buffer before final addition of 1 mL of Ac buffer at which point the postinfection time was considered to be 0 h. The infected protozoa was then incubated at 25°C and samples harvested at 72 h and 96 h. For microscopy, protozoan cells were gently resuspended with a pipette equipped with a 1000 *μ*L tip and ∼100 *μ*L was pipetted on a glass slide, covered with a cover slip and sealed with molten 2% agarose, and imaged/processed as described above.

## Results

### *Legionella pneumophila* colonization of *C. elegans*

**Figure 1:**
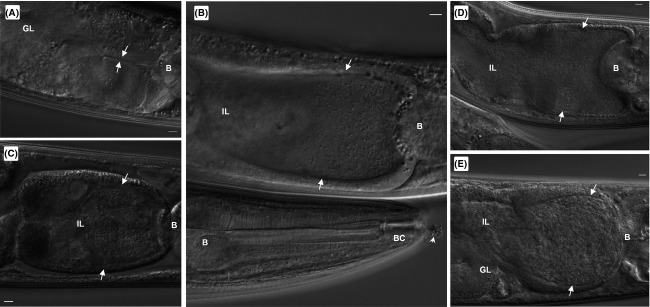
*Legionella pneumophila* colonizes the *Caenorhabditis elegans* intestinal lumen. Representative DIC images of N2 nematodes fed live Lp02 over time: (A) 2 days p.i.; (B) 3 days p.i.; (C) 4 days p.i.; (D) 6 days p.i.; and (E) 8 days p.i. Note also that the integrity of the intestinal lumen remains intact despite the progressive distension of the intestinal lumen due to the accumulation of colonized bacteria featuring typical rod-shaped morphology (white arrows). Anatomical features indicated in the microscopic images include the terminal bulb (B), buccal cavity (BC), intestinal lumen (IL) and the gonadal loop (GL). Scale bar represents 5 *μ*m in all panels.

It was previously reported that nematodes fed with live *L. pneumophila* Lp02 bacteria resulted in shortened nematode life span due to bacterial colonization of the intestinal lumen as demonstrated by microscopy of fluorescent protein-tagged Lp02 and quantification of the accumulated bacterial mass over time (Brassinga et al. 2010). However, at that time fluorescence microscopy did not detect intracellular Lp02 bacteria within the tissues of *C. elegans* nematodes leading to the presumption that the intestinal lumen provided the required environment and/or nutrients to support bacterial replication and differentiation (Brassinga et al. 2010). Recent reports have detailed the inhibitory effects of high levels of plasmid-borne GFP expression on the virulence of the intracellular pathogen *Salmonella enterica* such that invasion of host cells was compromised (Knodler et al. 2005; Clark et al. 2009). Since fluorescent microscopic studies were done in Brassinga et al. (2010) with plasmid-borne fluorescent protein expression, we wanted to follow bacterial colonization of *C. elegans* using differential interference contrast (DIC) microscopy to confirm similarities, and if any, differences with the previous studies. As observed in the Brassinga et al. (2010) study, Lp02 colonization of the intestinal tract progressed over time with marked intestinal distension noted by 3 days postinfection and onwards (Fig.1). It was noted that the intestinal epithelium remained intact during this colonization, but the intestinal lumen, anchored by attachment to the pharyngeal and rectal valves, expanded to accommodate the mass of replicating bacteria (Altun and Hall 2009). Constipation, caused by swelling and occlusion of the rectal opening, occurs early in colonized nematodes and remains constant for the duration of the life span of the nematodes. Constipation hinders the expulsion of bacteria via rhythmic peristalsis motion allowing for accumulation of bacterial mass in the intestine via replication (Avery and Thomas 1997; Brassinga et al. 2010). Rectal swelling and resultant constipation has been reported elsewhere as a consequence of pathogenic bacteria adhering to the rectal cuticle invoking a localized immune response mediated by the ERK MAP kinase pathway (Hodgkin et al. 2000; Nicholas and Hodgkin 2004; Irazoqui et al. 2010). While suggestive, the activation of the ERK MAP kinase pathway in response to colonization by *L. pneumophila* in *C. elegans* nematodes require further investigation to ascertain the participation of this pathway.

### LCVs in *C. elegans* pseudocoelomic cavity and gonadal tissue

**Figure 2:**
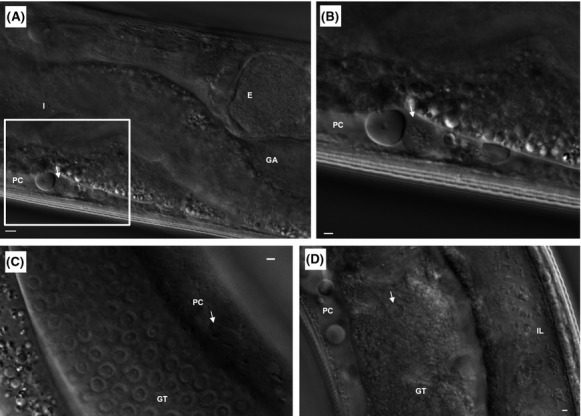
Formation of *Legionella*-containing vacuoles (LCVs) in *Caenorhabditis elegans* tissue and fluids. Representative DIC still micrographic images of spherical structures containing rod-shaped and/or coccoid shaped bacteria similar to LCVs in N2 nematodes fed live Lp02. (A and C) Spherical structures (indicated by white arrow) containing rod-shaped bacteria in the pseudocoelomic cavity (PC) of a nematode 2 days p.i. Note that the distinct structure of the intestine (I), and the distinct U-loop structure of the gonad arm (GA) (distal part is shown) containing the developing oocytes (not shown), the spermatheca (not shown) and embryos (E) (one is shown). Scale bar is 5 *μ*m. (B) Magnification of inset box in (A). Scale bar is 2 *μ*m. (D) Spherical structure (indicated by white arrow) containing coccoid-shaped bacteria within the gonadal tissue (GT) of a nematode 6 days p.i. Note the rod-shaped bacteria in the intestinal lumen (IL). Scale bar is 2 *μ*m. Still images in (A–C) are taken from Video S1, and still image in (D) taken from Video S2.

In addition to the bacterial colonization of the intestinal lumen, vacuoles containing nonmotile rod-shaped bacteria (presumably Lp02) were observed in the pseudocoelomic cavity first appearing 2 days postinfection (Fig.2A–C; Video S1A–C). Interestingly, vacuoles containing motile coccoid-shaped bacteria were also observed in the pseudocoelomic cavity as well as in the gonadal tissue in abundance by 6–7 days postinfection (Fig.2D; Video S2). It should be noted that these vacuoles containing bacterial forms were not previously observed in the Brassinga et al. (2010) study most likely due to high fluorescent protein levels obscuring or hindering vacuole formation. Furthermore, these vacuoles containing bacterial forms, in particular those vacuoles containing motile coccoid-shaped bacteria are morphologically similar to LCVs formed in *Legionella*-infected protozoa when nearing completion of its developmental lifecycle (Video S3). Vacuoles containing motile coccoid-shaped bacteria were also observed in the uterus and in the vulval region, but not in the vulval muscles, in the range of 4–7 days postinfection (data not shown). Furthermore, these bacteria-filled vacuoles were commonly observed in the fluidic material and/or viscera extruded from the vulva entry (Video S4). It should be noted that while great care was taken when preparing the colonized nematodes for imaging, extrusion of the material was not due to egg-laying since these animals no longer contained fertilized embryos at this time point in development, but appears to be a part of the pathologic process due to excessive fluid retention in the pseudocoelom of the infected host as noted in the Brassinga et al. (2010) study. Fluid extrusion from the vulva was frequently observed in dying nematodes on assay plates or in situ when mounted for imaging. In summary, formation of these vacuoles containing bacterial forms are specific to nematodes colonized with live Lp02 as these vacuoles were not observed in nematodes fed heat-killed Lp02 or in nematodes fed live and heat-killed OP50 (data not shown).

To further confirm whether these bacterial forms in vacuoles are in fact Lp02, a GFP-producing Lp02 strain was used. However, to circumvent the potential virulence attenuation effects by high levels of plasmid-borne GFP expression, a chromosomally encoded GFP expressing strain was generated for which levels of GFP would not likely interfere with virulence as shown previously with *S. enterica* Typhimurium (Clark et al. 2009). Specifically, a chromosome-integrated P_*magA*_::*gfpmut3* reporter construct strain was created which exhibited lower but developmental stage-specific GFP expression. Expression of *magA* is upregulated late in the *L. pneumophila* intracellular developmental cycle and lack of the encoded MagA 20 kDa product by genetic deletion did not appear to alter the virulence traits of Lp02 (Hiltz et al. 2004). Thus, it was decided to replace *magA* with *gfpmut3* via allelic genetic exchange to take advantage of the stage-specific upregulation in promoter activity as an indicator of cyst biogenesis. Also, because the nematode intestine naturally autofluoresces in the green/yellow channels (Clokey and Jacobsen 1986; Sifri et al. 2005), the transgenic nematode strain *glo-3*(*kx94*) was employed which featured reduced autofluorescence to facilitate detection of colonized Lp02 P_*magA*_::*gfpmut3* bacteria (Rabbitts et al. 2008) (Figure S2).

**Figure 3:**
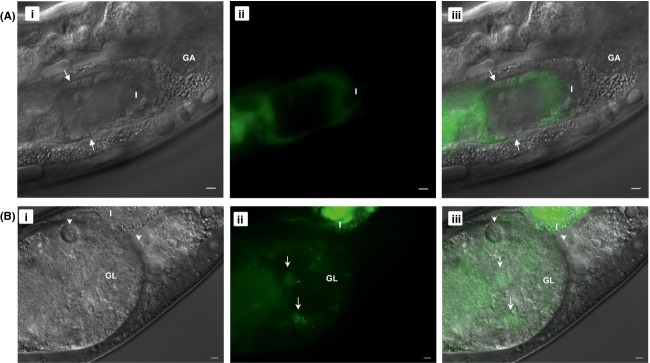
Intestinal and extra-intestinal locations of GFP-tagged *Legionella pneumophila* in *Caenorhabditis elegans*. Microscopic images of *glo-3*(*kx94*) nematodes fed with Lp02 P_magA_::*gfp* strain for (A) 5 days and (B) 6 days. Note the fluorescent rod-shaped bacteria (white arrow) in the intestine in (A) and forming a highly fluorescent bacterial mass in the intestine in (B). Note also the fluorescent (white arrows) and nonfluorescent (white arrowheads) coccoid-shaped bacteria in vacuoles (i.e., *Legionella*-containing vacuoles) in (B) of which only one is visible due to differing focal planes. Panels (i–iii) represent DIC, green and merge channels. Scale bar is 5 *μ*m. Still images in (B) taken from Video S5.

Bacterial colonization of the intestinal tract of *glo-3*(*kx94*) nematodes with Lp02 P_*magA*_::*gfpmut3* proceeded as observed with Lp02 in this study but as expected with a fluorescence intensity lower than previously observed with the plasmid-borne fluorescent protein expression of colonizing Lp02 in the Brassinga et al. (2010) study (Fig.3A). Vacuoles containing motile coccoid-shaped bacterial forms found in the pseudocoelomic cavity (data not shown) and gonadal tissue (Fig.3B, Video S5) were also fluorescent in the green channel confirming the identity of these bacterial forms as Lp02. It should be noted that only some, but not all of the motile coccoid-shaped bacterial forms, were fluorescent suggesting that the cyst forms became metabolically dormant and no longer expressed GFP protein upon completion of cyst biogenesis as detailed in Hiltz et al. (2004) (Fig.3B, Video S5). The vacuoles containing the motile coccoid-shaped bacterial forms, henceforth referred to as LCVs, are distinct in size, motility, and structure when compared to sperm contained within the spermatheca section of the *C. elegans* reproductive tract (Video S6).

### Formation of LCVs in the gonadal tissue is Dot/Icm dependent

**Figure 4:**
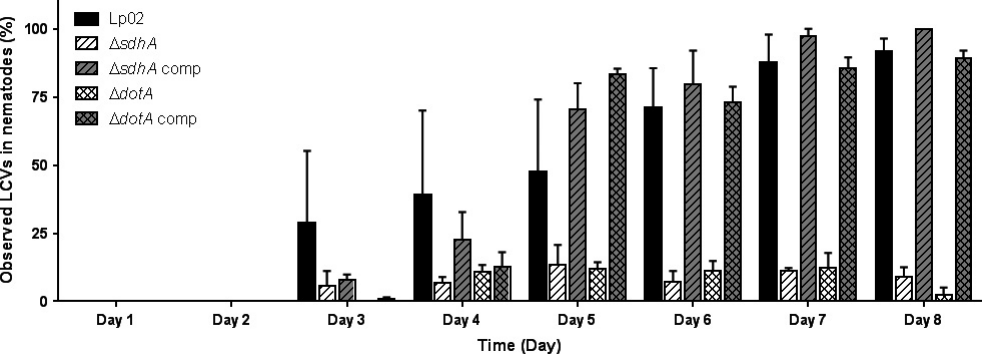
Presence of *Legionella*-containing vacuoles (LCVs) in the *Caenorhabditis elegans* gonadal tissue is dependent on the *Legionella pneumophila* Type IV Dot/Icm system. Enumeration of LCVs in the gonadal tissue of N2 nematodes fed wild-type Lp02, Δ*sdhA*, Δ*dotA* mutant and complemented strains plotted over time (8 days). For each strain and timepoint, 20–30 nematodes were examined by DIC microscopy. Three independent experiments were performed and bars represent mean values with SEM.

After internalization of *L. pneumophila* by a macrophage, manipulation of the endocytosis pathways to subvert the phagosome-lysosome pathway and establish a replicative niche is dependent on the Dot/Icm system (Isberg et al. 2009; Hubber and Roy 2010; Escoll et al. 2013). Lack of DotA, an essential component, renders the Dot/Icm system dysfunctional with the result that the LCV is formed but is nonreplicative eventually fusing with lysosomes (Berger and Isberg 1993; Berger et al. 1994). Likewise, lack of SdhA, normally required to maintain the integrity of the LCV, results in the instability of the LCV triggering the macrophage innate immune pathways and cell death (Creasey and Isberg 2012). Because a substantial amount of LCVs was observed in the gonadal tissue of the colonized nematodes, we decided to use DIC microscopy to precisely detail the timeline of the appearance and quantity of LCVs in the gonadal tissue as well as determine if the formation of LCVs was dependent on a functional Dot/Icm system or SdhA (Fig.4). First appearance of LCVs in the gonadal tissue of nematodes colonized with Lp02 was observed at 3 days postinfection with variance in frequency which decreased as the number of LCVs increased by 6 days postinfection onwards. Interestingly, the number of LCVs dropped significantly in nematodes colonized with Δ*dotA* or Δ*sdhA* mutant strains but increased to wild-type levels when complemented. It should be noted that the number of LCVs in the pseudocoelomic cavity of nematodes colonized with Δ*dotA* or Δ*sdhA* mutant strains were marginally decreased in comparison to levels observed in nematodes colonized with Lp02 (data not shown). Thus, likewise to their functional roles in macrophages, DotA and SdhA appear to be required for the formation of viable LCVs in the *C. elegans* gonadal tissue.

### Absence of SdhA and DotA prolongs the lifespan of *C. elegans*

**Figure 5:**
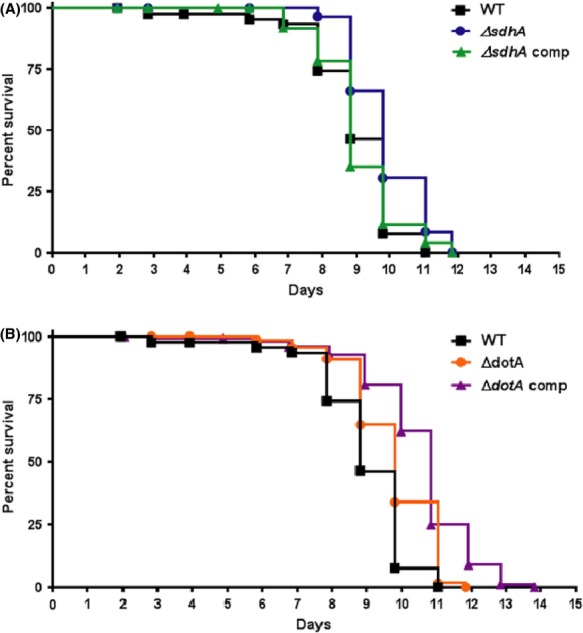
Survival of nematodes is affected by the *Legionella pneumophila* Type IV Dot/Icm system. Kaplan–Meier survival plot of *Caenorhabditis elegans* N2 nematodes fed: (A) wild-type Lp02 (squares, *n *=* *43), Δ*sdhA* mutant (circles, *n *=* *58) and Δ*sdhA* complemented (triangles, *n *=* *33) strains. *P *=* *0.004 and *P *=* *0.0016 by pairwise comparison by the log-rank test of Δ*sdhA*- and Lp02-fed nematodes, and Δ*sdhA*- and Δ*sdhA* comp-fed nematodes, respectively. Pairwise comparison between Lp02- and Δ*sdhA* comp-fed nematodes was found to be statistically insignificant (*P *=* *0.9805); and (B) wild-type Lp02 (squares, *n *=* *43), Δ*dotA* mutant (circles, *n *=* *63) and Δ*dotA* complemented (triangles, *n *=* *90). *P *<* *0.0001 by pairwise comparison by the log-rank test of Δ*dotA*- and Lp02-fed nematodes, and Δ*dotA*- and Δ*dotA* comp-fed nematodes. *P *=* *0.0010 by pairwise comparison by the log-rank test of Lp02- and Δ*dotA*-fed nematodes.

To ascertain if the lack of SdhA or DotA affected the survival of *C. elegans* on an organismal level, *C. elegans* survival assays were conducted with nematodes fed Δ*dotA* or Δ*sdhA* mutant and complemented strains along with the wild-type Lp02 included as a positive control. The survival rate of nematodes fed the Δ*sdhA* strain significantly increased in comparison to nematodes fed Lp02 (Fig.5A). When fed the Δ*sdhA* complemented strain, the survival rate reverted to that observed for nematodes fed Lp02 (Fig.5A). Likewise, the same trend was observed for nematodes fed the Δ*dotA* strain; however, the survival rate of nematodes fed the Δ*dotA* complemented strain did not revert to that of nematodes fed Lp02 (Fig.5B). While uninduced expression of the *dotA* complement plasmid pKB9 was sufficient to compensate for lack of chromosomally expressed DotA in *L. pneumophila* in infected macrophages, high levels of DotA achieved under inducing conditions leads to loss of viability (Roy et al. 1998). As the control of *dotA* expression from pKB9 is not precise with respect to achieving wild-type levels, it is possible that inappropriate expression levels of DotA prevent the rescue of the wild-type phenotype in *C. elegans*.

### Activation of germline apoptosis may be inhibited by SidF, but not SdhA

**Figure 6:**
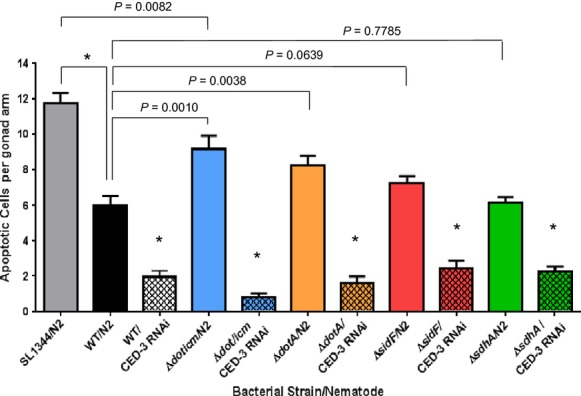
Elevated levels of germline apoptosis in the *Caenorhabditis elegans* gonadal tissue are dependent on the *Legionella pneumophila* Type IV Dot/Icm system. Number of corpse (i.e., apoptotic) cells counted per gonad (*n *=* *20–30) in *ced-1::gfp* (MD701) and RNAi-treated (*ced-3*) *ced-1::gfp* (MD701) nematodes fed wild-type Lp02, Δ*dot/icm*, Δ*dotA*, Δ*sdhA*, and Δ*sidF* mutant strains as well as *S*. Typhimurium SL1344. Bars are mean values with SEM. *P* values obtained by unpaired two-tailed student’s test with Welch’s correction; *P <* *0.0001.

In *C. elegans*, susceptibility to Lp02 is influenced by innate immune responses governed by the p38 MAPK and insulin-like DAF-2 signaling pathways as loss-of-function mutant nematodes exhibited an immunocompromised phenotype (Brassinga et al. 2010). Activation of the p38 MAPK pathway, functionally orthologous to the macrophage MAPK pathway, triggers the activation of the downstream germline apoptosis (i.e., programmed cell death [PCD]) pathway (Aballay and Ausubel 2001; Kinchen and Hengartner 2005; Gartner et al. 2008). As key components of the core apoptotic machinery CED-9/CED-4/CED-3 are homologous to the mammalian BCL2/APAF-1/CASPASE, it was previously determined that a functional *L. pneumophila* Dot/Icm system bestowed an antiapoptotic effect on the PCD pathway (Brassinga et al. 2010). However, the identity of the Dot/Icm-mediated substrates that interacted with the PCD pathway was not known. In *L. pneumophila*-infected macrophages, caspase-mediated apoptosis is blocked by the interaction of effector molecule SidF with proapoptotic proteins BNIP3 and Bcl-rambo (Banga et al. 2007). To determine if the substrate SidF is involved in inhibiting apoptosis mediated by the PCD pathway in *C. elegans*, apoptotic cells were enumerated in transgenic *P*_*lim-7*_*ced-1::gfp* nematodes fed wild-type Lp02 and Δ*sidF* mutant strains. CED-1, a transmembrane receptor expressed on the surface of somatic sheath cells, is essential for the engulfment of apoptotic germ cells and thus translational fused CED-1:GFP provides a convenient visual marker of apoptotic cells for enumeration (Hedgecock et al. 1983; Conradt and Horvitz 1999; Schumacher et al. 2005). Because SdhA has been shown to maintain the integrity of the vacuole rather than interact with the macrophage apoptosis pathway (Creasey and Isberg 2012), the Δ*sdhA* strain was included as a negative control substrate as it is expected that the apoptotic cell levels will not be altered in comparison to levels achieved in nematodes colonized with Lp02. As an additional control, nematodes with RNAi-mediated downregulation of the caspase *ced-3* were included to verify that apoptosis was activated by CED-3. As previously reported in Brassinga et al. (2010), apoptotic cell levels were significantly elevated in *P*_*lim-7*_*ced-1::gfp* nematodes fed the Δ*dotA* strain that features a dysfunctional Dot/Icm system (Fig.6). Apoptotic cell levels were likewise elevated in *P*_*lim-7*_*ced-1::gfp* nematodes fed the Δ*dot/icm* strain in which the *icm/dot* loci are genetically deleted (Fig.6). The absence of the substrate SidF, but not SdhA, marginally increased apoptotic cell levels suggesting that other Dot/Icm substrate(s) may interact directly with CED-3 to inhibit activation of apoptosis (Fig.6).

### Internalization and morphological differentiation of *L. pneumophila* Lp02 bacteria in *C. elegans* tissue

**Figure 7:**
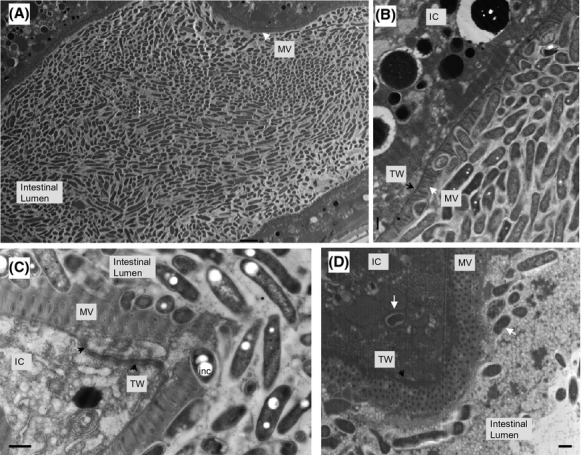
*Legionella pneumophila* invades the *Caenorhabditis elegans* intestinal cells. Representative transmission electron microscopy images were obtained from samples of nematodes fed *L. pneumophila* Lp02 for 6 days. (A) Longitudinal section of colonized intestine. Note the typical rod-shaped morphology of the bacteria within the intestinal lumen defined by the microvilli (MV) intestinal epithelium lined by the MV. Scale bar is 2 *μ*m. (B) Embedding of the bacteria within the MV on the apical surface of the intestinal cell (IC) supported by the terminal web (TW). Note the poly-*β*-hydoxybutyrate inclusion bodies (white spots) within some of the bacterial forms. Scale bar is 500 nm. (C) Disruption of the electron dense intermediate filaments of the TW from the MV on the apical surface of the IC. Note the presence of the bacterial form with poly-*β*-hydoxybutyrate inclusion (inc) bodies embedded in the MV. Scale bar is 500 nm. (D) Internalization of a bacterial form (white arrow) within the IC. Note that the shape and size of the internalized bacterial form is similar to bacterial forms (white arrow) present within the intestinal lumen. Also note that the MV with the supporting TW appears to have re-formed. Scale bar is 500 nm.

The presence of LCVs in the *C. elegans* gonadal tissue poses the question of how Lp02 bacteria are able to gain access to the gonadal tissue from the colonized intestinal tract. Additionally, does the bacteria differentiate while enroute to the gonadal tissue or remain static in their morphological form when leaving the colonized intestinal tract? To answer these questions, TEM was employed in order to track the probable progress of the bacteria from the colonized intestinal tract to the gonadal tissue and analyze the ultrastructural features to determine the developmental stage of *L. pneumophila* forms based on previously defined morphological criteria. A longitudinal TEM section illustrates the dense packing of Lp02 bacteria in the intestinal lumen of a colonized nematode (Fig.7A). A magnified focused view on a section of the intestinal epithelium shows that the majority of Lp02 bacteria in the intestinal lumen feature a mixture of replicative, stationary phase forms (some featuring PHBA inclusion bodies) (Fig.7B) (Faulkner and Garduño 2002; Brassinga et al. 2010). In addition, embedment of Lp02 bacteria were frequently observed within the microvilli that extend from the apical surface to form a brush border suggesting that Lp02 bacteria have the ability to penetrate the glycocalyx electron-lucent coating of highly modified glycoproteins termed glycocalyx covering the microvilli (Fig.7B) (Lehane 1997; McGhee 2007).

**Figure 8:**
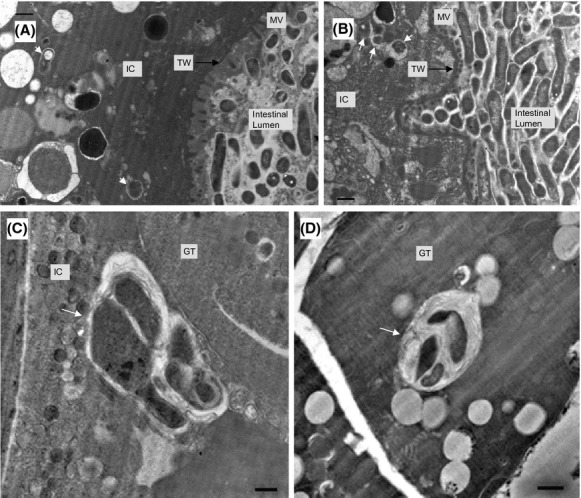
Morphological differentiation of internalized *Legionella pneumophila* bacterial forms. Representative transmission electron microscopy images were obtained from samples of nematodes fed *L. pneumophila* Lp02 for 6 days. (A and B) Internalized bacterial forms (white arrow) within intestinal cells (IC) with microvilli (MV) on the apical surface supported by the terminal web (TW). Note that the bacterial membrane is more defined due to progressive formation of multiple layers. (C) Irregular- shaped transitional bacterial forms (white arrow) inside an IC next to the gonadal tissue (GT) (i.e., gonad arm). (D) Presence of a well-defined *Legionella*-containing vacuoles, containing irregular-shaped transitional forms and membrane whorls (indicated by white arrow), in a GT cell of the gonad arm. Membrane whorls most likely represent eukaryotic membrane fragments. Scale bar is 500 nm for all panels.

The embedded Lp02 bacteria appear to disrupt the underlying terminal web, a strong cytoskeletal network consisting largely of intermediate filaments anchoring the microvilli (McGhee 2007), gaining access to the intestinal cell (Fig.7C). Indeed, single internalized bacterial forms with typical gram-negative ultrastructure features are observed within a tight vacuole in the intestinal cell (Fig.7D). Furthermore, single and grouped transitional bacterial forms are also observed to be internalized within tight vacuoles in intestinal cells (Fig.8A and B). Transitional bacterial forms are intermediate forms prior to the formation of cyst forms and are characterized to feature wavy well-defined membranes incipient of a thick layer, and may sometimes feature PHBA inclusions. Adjacent to and in the gonadal tissue, tight vacuoles containing multiple irregular shaped bacterial forms with multiple membrane laminations were observed to be morphologically similar to LCVs containing differentiating *L. pneumophila* cyst forms in protozoa and nonphagocytic cells as reported elsewhere (Fig.8C and D) (Faulkner and Garduño 2002; Berk et al. 2008). This suggests that replication occurred within the vacuole until nutritional sources are depleted triggering the process of replicative forms transitioning into cyst forms. Taken together, these ultrastructural observations suggest that *L. pneumophila* Lp02 appears to have the ability to transverse the intestinal epithelium into the intestinal cell from the colonized intestinal tract as well as differentiate to cyst forms. It should be noted that despite repeated attempts, it was not possible to identify Lp02 bacteria within the *C. elegans* tissue on the basis of immunogold labeling due to the thickness of the tissue in the fixed sections of colonized nematodes. Conversely, Lp02 bacteria within the intestinal tract were readily immunogold labeled due to the lack of interfering tissue when sectioning the colonized nematodes in a longitudinal manner (Figure S1). Thus, analyses of the ultrastructural features of bacterial forms via TEM imaging constituted the best available approach to identify the multiphasic forms of *L. pneumophila*.

### The endocytic factor RME-1 is involved in the uptake of LCVs into the gonad

*Caenorhabditis elegans* do not possess a true vascular system and therefore rely heavily on the fluid-filled pseudocoelomic cavity and exocytosis/endocytosis pathways to move nutrients from the intestinal lumen to other organs (Sato et al. 2014). Oocytes in the gonad require nutrients in the form of yolk to facilitate their development. Yolk, a complex of proteins, cholesterol, phospholipids, and triglycerides, is synthesized in the intestinal cells and secreted into the pseudocoelomic cavity for uptake via the oocyte endocytosis pathway (Kimble and Sharrock 1983; Sharrock et al. 1990; Matyash et al. 2001; Fares and Grant 2002). One characterized component of the endocytosis pathway is the endocytic factor RME-1 that is localized to the basolateral recycling endosomes in intestinal cells playing an important role in receptor-mediated endocytosis (Grant et al. 2001). Lack of RME-1 not only results in the accumulation of grossly enlarged endosomes filled with endocytosed fluid in the pseudocoelomic cavity and basolateral compartments of intestinal cells, but also blocks yolk uptake by oocytes (Grant and Hirsh 1999).

**Figure 9:**
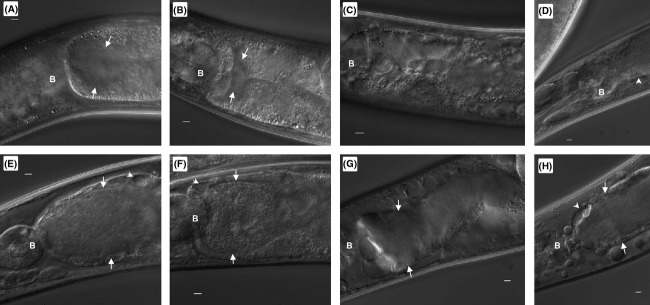
Intestinal colonization of *Legionella pneumophila* in endocytosis-defective *rme-1*(*b1045*) nematodes. Representative DIC images of *rme-1*(*b1045*) nematodes fed live *Escherichia coli* OP50 over time: (A) 2 days p.i.; (B) 4 days p.i.; (C) 6 days p.i.; (D) 8 days p.i.; and live Lp02 over time (E) 2 days p.i.; (F) 4 days p.i.; (G) 6 days p.i.; (H) 8 days p.i. Note that the integrity of the intestinal lumen remains intact despite the progressive distension of the intestinal lumen due to the accumulation of colonized bacteria (white arrows) and progressive vacuolation of the intestinal epithelium (white arrowheads) due to inefficient recycling of the oocyte endosome receptor in the absence of RME-1. Anatomical features indicated in the microscopic images include the terminal bulb (B). Scale bar represents 5 *μ*m in all panels.

To determine if RME-1 is involved in translocating LCVs to the gonadal tissue, loss-of-function *rme-1*(*b1045*) nematodes were fed wild-type Lp02 and *E. coli* OP50 (control) and monitored for colonization by these strains as well as formation of LCVs over time via DIC microscopy. In *rme-1*(*b1045*) nematodes fed *E. coli* OP50, a small amount of bacterial colonization in the intestinal tract was observed by 2 and 4 days postinfection (Fig.9A and B). While the bacterial colonization did not cause further intestinal distension in *rme-1*(*b1045*) nematodes by 6 and 8 days postinfection, numerous endosomes are observed in the pseudocoelomic cavity and basolateral compartment of the intestinal cells as typified by this loss-of-function mutation (Fig.9C and D). Colonization and intestinal distension progressed in *rme-1*(*b1045*) nematodes fed Lp02 as previously observed with N2 nematodes fed Lp02 (Figs.1, 9E–H). Interestingly, there was a consistent absence of LCVs in the gonadal tissues throughout the 8 days of monitoring *rme-1*(*b1045*) nematodes colonized with Lp02. Conversely, while LCVs were seen in intestinal cells, a higher than normal levels of LCVs were observed in the pseudocoelomic cavity of which a subset were found in proximity to the gonadal tissue. This result suggests that lack of RME-1 results in the accumulation of LCVs in pseudocoelomic cavity and prevents uptake of LCVs into the gonadal tissue.

### Lack of RME-1 prolongs the life span of *rme-1*(*b1045*) nematodes colonized with Lp02

**Figure 10:**
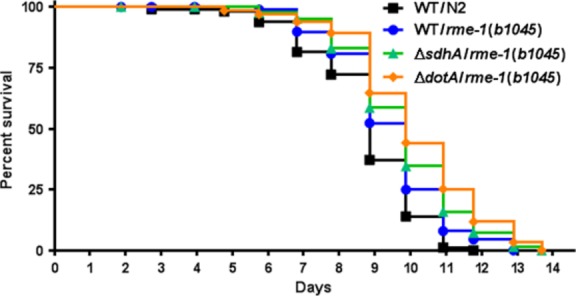
Survival of endocytosis-defective nematodes fed *Legionella pneumophila* is prolonged. Kaplan–Meier survival plot of *Caenorhabditis elegans* N2 nematodes fed wild-type Lp02 (squares, *n *=* *87), and *rme-1*(*b1045*) nematodes fed wild-type Lp02 (circles, *n *=* *88), Δ*sdhA* (triangles, *n *=* *77) and Δ*dotA* (diamonds, *n *=* *63) strains. Pairwise comparison by the log-rank test of N2 nematodes fed Lp02 versus *rme-1*(*b1045*) nematodes fed Δ*sdhA* and Δ*dotA* strains is *P *< 0.001, *P *=* *0.0072 for N2 nematodes fed Lp02 versus *rme-1*(*b1045*) nematodes fed Lp02, and *P *=* *0.0046 for *rme-1*(*b1045*) nematodes fed Lp02 versus *rme-1*(*b1045*) nematodes fed Δ*dotA*. Pairwise comparison of *rme-1*(*b1045*) nematodes fed Lp02 and *rme-1*(*b1045*) nematodes fed Δ*sdhA* was found to be statistically insignificant (*P *=* *0.1366).

The absence of LCVs in the gonadal tissue of Lp02-colonized *rme-1*(*b1045*) nematodes prompted us to investigate if the lack of RME-1 also affected the lifespan. To answer this question, *rme-1*(*b1045*) nematodes were fed wild-type Lp02, Δ*sdhA* and Δ*dotA* mutant strains along with N2 nematodes fed wild-type Lp02 as a control. Similar to what was observed with N2 nematodes colonized with *sdhA* or Δ*dotA* mutant strains, the life span of *rme-1*(*b1045*) nematodes colonized with Lp02 was prolonged in comparison to the survival rate of N2 nematodes colonized with Lp02 (Fig.10). In addition, the life span of *rme-1*(*b1045*) nematodes was further prolonged when colonized with Δ*sdhA* or Δ*dotA* mutant strains (Fig.10). Taken together, these results suggest that uptake and establishment of LCVs in the gonadal tissue is an integral part of the Dot/Icm system-dependent disease pathology of nematodes colonized with *L. pneumophila* Lp02.

## Discussion

Protozoa have long been regarded as the sole natural hosts that support the intracellular lifestyle of *L. pneumophila* (Fields 1996; Hägele et al. 2000; Lau and Ashbolt 2009; Newton et al. 2010; Escoll et al. 2013). This association promotes the co-evolution of traits allowing *L. pneumophila* to effectively parasitize protozoa in the natural environment (Albert-Weissenberger et al. 2007). Protozoa are ubiquitous in the soil environment along with a diverse range of organisms; however, the possibility that ecological relationship(s) may exist between *L. pneumophila* and a nonprotozoan host has not been well examined. The potential role of bacterivorous soil nematodes as an environmental reservoir of *L. pneumophila* was investigated in a previous study (Brassinga et al. 2010). Viable bacteria were able to survive in nematodes in a simulated soil environment devoid of nutrients required to support bacterial replication ex vivo. Because *L. pneumophila* is a facultative intracellular bacterium, we expanded our investigations on *L. pneumophila*-infected nematodes to include focused TEM and DIC microscopic approaches to definitively determine whether bacterial invasion of nematodal tissue from the colonized intestinal lumen was possible. Here, we report the findings of LCVs-containing replicative forms and differentiated cysts in the pseudocoelomic cavity and gonadal tissue in *C. elegans* nematodes colonized with Lp02. To our knowledge, this is the first report of a host other than the protozoa or HeLa cell model that appears to support the full morphological differentiation of *L. pneumophila* into cyst forms. Our findings strengthen the proposal that nematodes could be an additional reservoir of *L. pneumophila* in the soil environment. This has novel implications to the epidemiology of human infections by *L. pneumophila* and is compatible with reports of individuals contracting Legionnaires’ disease after exposure to potting soil (Casati et al. 2009; Whiley et al. 2011).

The close proximity of the gonadal tissue to the colonized intestinal lumen may have provided the means of opportunity for Lp02 bacteria to gain access to the gonadal tissue via the oocyte endocytosis pathway. The oocyte endocytosis pathway is responsible for the internalization of large quantities of yolk proteins and associated lipids produced by the intestinal cells for uptake into maturing oocytes through a clathrin-mediated endocytosis (Grant and Sato 2006). To date, 11 *rme* (receptor-mediated endocytosis defective) genes (*rme-1* to *rme-11*) have been identified to be required for various steps in endocytic transport (Grant and Hirsh 1999). RME-1 and RME-8, found in the cytoplasm of most *C. elegans* cells, has been well characterized as regulators of the oocyte endocytosis pathway. RME-1 is an EH domain protein associated with the recycling of endosome receptors, and RME-8 is a J-domain protein of which implied functional role is to interact with heat-shock protein 70 family members to uncoat the clathrin-coated vesicles through a cytoplasmic action (Brodsky et al. 2001; Grant et al. 2001; Zhang et al. 2001). RME-2 is a receptor specifically expressed in oocytes that binds to yolk material for uptake into clathrin-coated pits (Grant and Sato 2006). The yolk protein YP170, characterized as a cholesterol-binding/transport protein related to human ApoB-100, is secreted basolaterally into the pseudocoelomic cavity and has been identified to be recognized by RME-2 for uptake (Grant and Hirsh 1999; Grant and Sato 2006). Other factors identified to be associated with the oocyte endocytosis pathway are endocytic Rab proteins RAB-5, RAB-7 and RAB-11 which are essential for the trafficking of yolk and yolk receptors (Grant and Hirsh 1999; Grant and Sato 2006).

In this study, RME-1 has been identified to be essential for establishment of LCVs in the gonadal tissue giving the first indication of the involvement of the oocyte endocytosis pathway in the formation of LCVs in *C. elegans* tissue. The invasion of *C. elegans* intestinal cells by Lp02 bacteria colonizing the intestinal lumen may be facilitated by Hsp60. It has been shown elsewhere that Hsp60 (encoded by *htpB*) arrayed on the surface of *L. pneumophila* bacteria promotes attachment and invasion of nonphagocytic eukaryotic cells as well as alter organelle trafficking (Garduño et al. 1998; Chong et al. 2009). Interestingly, yolk storage vesicles secreted from the intestinal cells are considered to be functional analogs of lysosomes with the exception that they lack hydrolytic activity (Schneider 1996; Fares and Grant 2002). To this end, a model illustrating the uptake and transport of LCVs in *C. elegans* is proposed in Figure1. The gonad comprises the gonad arm that contains the stem cells and the oocytes prior to fertilization, and the egg-laying apparatus, located just distal to the spermatheca where fertilization occurs, that includes the uterus, vulva, and their corresponding muscles involved in expelling embryos (Altun and Hall 2009). Intestinal cells bordering the colonized intestinal lumen are invaded by Lp02 bacteria that become encased in vacuoles, perhaps similar to yolk material, which are then released from the basal surface of the intestinal cells into the pseudocoelomic cavity and are putatively taken up into developing oocytes by RME-2-mediated endocytosis. LCVs are observed to contain replicative forms as well as differentiated forms indicating that proliferation and biogenesis of cysts are achieved. Studies are currently underway to determine if RME-2 and RME-4 as well as RAB-5 and RAB-35 are involved due to their previously described function in endocytosis and recycling at the clathrin-coated pit process (Fig.1) (Brodsky et al. 2001). Taken together, the similarities with the well-characterized steps of endosome-lysosome fusion in *Legionella*-infected macrophages and protozoa can be related to the oocyte endocytosis pathway in *C. elegans*.

**Figure 11:**
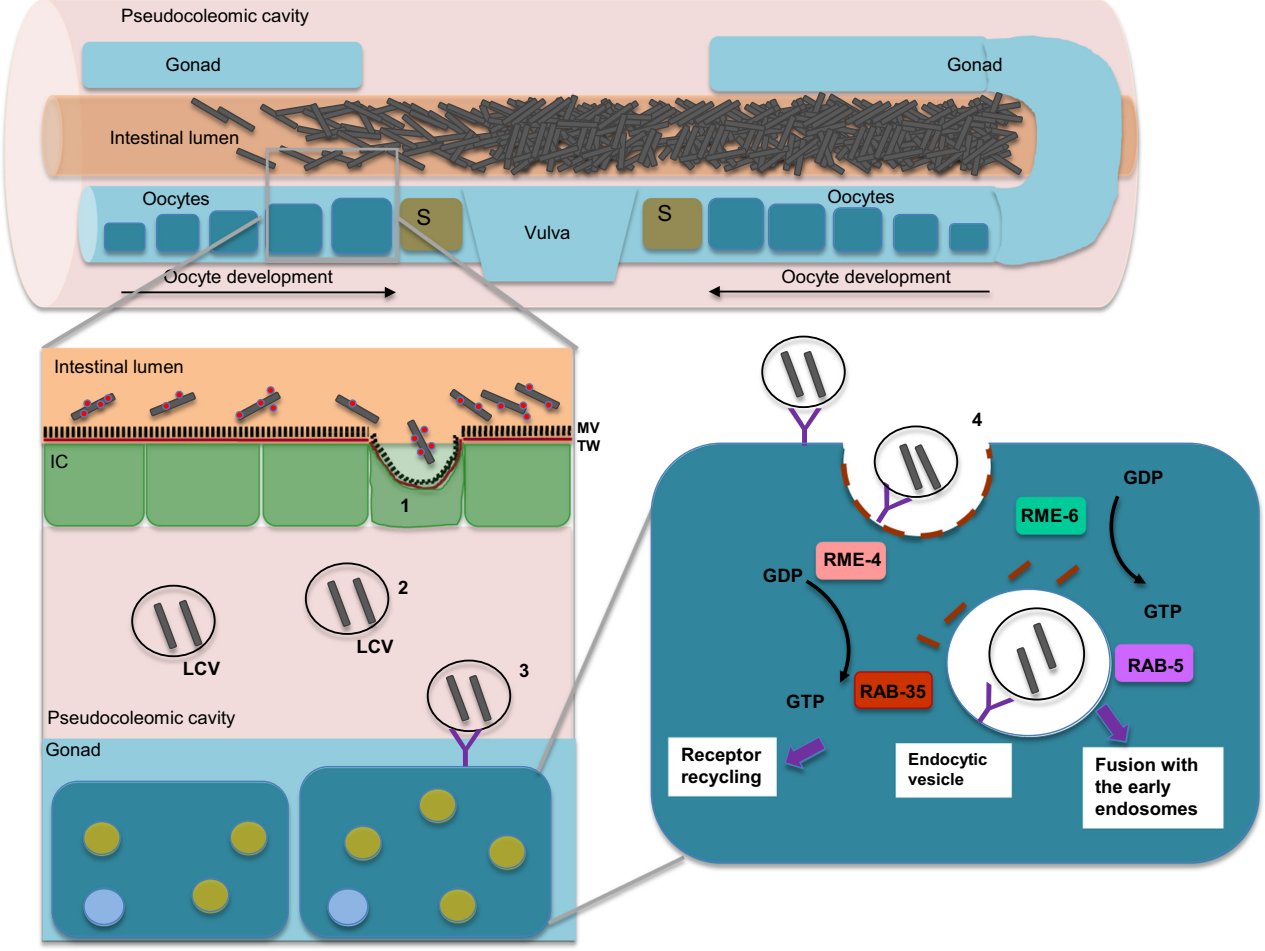
Model of the passage of *Legionella pneumophila* from the colonized intestinal lumen to the gonadal tissue via the oocyte endocytosis pathway. Schematic representation of the anatomical mid-section of the *Caenorhabditis elegans* nematode colonized with *L. pneumophila* (grey-brown rods). Note that the pseudocoelomic cavity fluid (pink) encompasses the reproductive organs (blue) that includes the gonads containing the maturing oocytes, spermatheca (S), uterus and vulva structures) and the intestinal lumen (orange) containing *L. pneumophila* bacteria (brown rods). The intestinal epithelium comprises the microvilli (MV, black brush border), terminal web (TW, red line) and intestinal cells (IC, green). The following mechanisms are proposed as illustrated in the enlarged section of the schematic: (1) bacteria invades the intestinal cell assisted by Hsp60 (red dots); (2) bacteria are enveloped into a vacuole formation (i.e., *Legionella*-containing vacuoles [LCVs]) that possibly includes yolk particles and secreted from the intestinal cell into pseudocoelomic cavity; (3) LCVs are recognized by the RME-2 receptor (purple “Y”); and (4) LCVs taken up by oocytes by a clathrin (brown dashes)-dependent endocytosis mechanism that involves RME-4, RME-6, RAB-5 and RAB-35 (adapted from Grant and Sato 2006). Note that the oocyte contains nucleic acid (solid blue circle) and that yolk granules (solid green circle) accumulate within the oocyte throughout the maturation or development process. Note that the schematics depicting *C. elegans* anatomical features and *L. pneumophila* bacteria are not to scale nor correctly proportioned.

Alteration of germline apoptosis levels as a result of bacterial colonization has been previously reported with *S*. Typhimurium, but not with *Pseudomonas aeruginosa*, indicating a pathogen-specific interaction (Aballay and Ausubel 2001). However, it was not immediately apparent the precise mechanisms by which *S*. Typhimurium affected germline apoptosis as no bacteria were observed near or in the gonadal tissues in spite of a colonized distended intestinal tract (Aballay et al. 2000). It should be noted that *S*. Typhimurium can invade nematodal tissue in nematodes carrying a loss-of-function Toll-like Receptor (TLR) homolog *tol-1* gene or the autophagic genes *bec-1* and *lgg-1* indicating that functional host defenses are required to prevent invasion by *S*. Typhimurium (Aballay and Ausubel 2001; Tenor and Aballay, 2008; Jia et al. 2009). A facultative intracellular pathogen, *S*. Typhimurium possesses two Type III Secretion Systems that mediate the secretion and translocation of effector molecules to remodel the cellular processes in order to establish *Salmonella*-containing vacuole in mammalian intestinal epithelial cells and macrophages (Agbor and McCormick 2011). Interestingly, activation of several effector molecules associated with virulence is dependent on cleavage by caspase-3 (Srikanth et al. 2010). The interaction with the host protein caspase-3 appears to be a common element between *S*. Typhimurium and *L. pneumophila*. While the absence of SidF did not completely alleviate repression of apoptosis in nematodes colonized with the Δ*sidF* mutant strain, it is quite possible that functional redundancy exists among Dot/Icm substrates targeting the same target in the host cell as the case is for other targets reported elsewhere (Dorer et al. 2006; Newton et al. 2010). This view is further supported by the result of significantly elevated germline apoptosis levels in nematodes colonized with the null Δ*dot/icm* mutant strain defective for translocation of all Dot/Icm substrates (Fig.6). Further studies are required to investigate the interactions between host defenses and Dot/Icm substrates in *C. elegans*.

The observed presence of LCVs in the gonadal tissue highlights the plausible evolutionary origin of the abilities and strategies employed by *L. pneumophila* to modulate and evade eukaryotic host cellular processes. It should be noted that nematodes are exposed to an excess of *L. pneumophila* when feeding ad libitum in plate-based assays which is generally not reflective of concentrations that free-living nematodes might encounter in the natural soil environment (Brassinga et al. 2010). Concentrations of *Legionella* in potting soils, organic composts and garden soils are in the range of 10^3^–10^8^ cfu per gram of soil (Hughes and Steele 1994; Casati et al. 2009). Indeed, successive generations of nematodes were able to survive for a long period of time in sterile soil seeded with *L. pneumophila* (Brassinga et al. 2010). Nematodes colonized with low levels of *L. pneumophila* may lead to a tolerant relationship in a similar fashion to the microsporidium *Nematocida parisii* parasitic relationship. Transmitted by the fecal–oral route, the fungal-related intracellular pathogen *N. parissi* invades *C. elegans* intestinal cells and rearranges the host cell cytoskeleton to establish a chronic infection for dissemination of spores into the environment (Troemel et al. 2008; Estes et al. 2011). Naturally occurring levels of *N. parisii* spore density in a typical soil environment have not been reported although chronically infected nematodes shed ∼10^4^ spores per nematode under experimental conditions implying a direct proportional relationship regarding the population densities of the host and obligate pathogen (Estes et al. 2011). It has been postulated that co-evolution has occurred between *C. elegans* and *N. parisii* to reduce virulence thereby protecting the health of the host while maximizing spore production and transmission (Estes et al. 2011; Balla and Troemel 2013). With respect to nematodes colonized with *L. pneumophila*, it is not clear how LCVs would be released back into the environment. The observation of LCVs in material extruded from the vulva suggests perhaps that dissemination of LCVs into the environment is achieved by the vulvar release of infected oocytes or embryos. Further studies employing nematodes exposed to low levels of *L. pneumophila* will need to be conducted to determine the extent of intestinal and gonadal colonization of bacteria to establish the parameters of the parasitic relationship.

## Supporting Information

**Figure S1.** TEM imaging of immunogold-labeled *Legionella pneumophila* colonizing the *Caenorhabditis elegans* intestinal lumen. A) Overall view of bacteria along the microvilli (MV) surface of an intestinal cell (IC). B) Transitional bacterial forms which are morphologically similar to either stationary phase forms or previously observed intermediate forms (Faulkner and Garduño 2002) were identified by characteristic morphological features such as wavy outer membranes and a fragmented cytoplasm (indicated by arrow). Scale bar represents 500 nm in all panels. It should be noted some gold particles (black particles) are offset from the bacterial membranes due to the fact that the gold particles are conjugated to secondary antibodies which are ∼2 nm in length. **Figure S2.** Autofluorescence of nematodes. Microscopic images of the posterior regions of A) *glo-3*(*kx94*) and B) N2 adult nematodes. Panels (i–iii) represent green, DIC, and merge channels, respectively. Note the prominent autofluorescence due to lipofuschin present in the gut granules in the wild-type N2 nematode which is significantly reduced in the *glo-3*(*kx94*) nematode.

**Video S1.** *Legionella*-containing vacuoles (LCV) in the pseudocoelomic cavity of *Caenorhabditis elegans* nematodes. DIC microscopic videos of a N2 *C. elegans* nematode fed live *Legionella pneumophila* Lp02 2 days postinfection. Video images correspond to Figure 1a–c. (A) A vacuole containing rod-shaped bacteria (indicated by white arrow) in the pseudocoelomic cavity adjacent to the intestinal tract. Scale bar is 5 *μ*m; and (B) enlargement of the area indicated by inset box in Figure 1a with focus on the *Legionella*-containing vacuole (indicated by white arrow). Scale bar is 2 *μ*m. (C) Vacuole containing rod-shaped bacteria (indicated by white arrows) in the pseudocoelomic cavity of the nematode adjacent to the gonad. Scale bar is 2 *μ*m.

**Video S2.** A *Legionella*-containing vacuole (LCV) in the gonadal tissue of a *Caenorhabditis elegans* nematode. DIC microscopic videos of a N2 *C. elegans* nematode fed live *Legionella pneumophila* Lp02 6 days postinfection. Video images correspond to Figure 1d. Note a vacuole containing motile coccoid-shaped bacteria (indicated by white arrow) in the gonadal tissue. Comparison of the size and shape of the bacteria found in the vacuoles versus bacteria in the intestinal lumen reveals distinct morphological differences.

**Video S3.** *Legionella*-containing vacuole (LCV) in *Acanthamoeba castellanii* protozoa. DIC microscopy video of an (A) uninfected protozoan cell and (B) infected protozoan cell with Lp02 *thyA* comp for 96 h at 25°C. Note the increased speed of coccoid-shaped bacteria in the LCV relative to protozoan cytoplasmic contents. The coccoid-shaped bacteria are similar in size, shape, and motility to the bacteria within LCVs in the infected nematode as observed in Video S2, S4 and S5. Scale bar is 2 *μ*m.

**Video S4.** *Legionella*-containing vacuoles (LCVs) in extruded material from the vulva. DIC microscopy video of extruded material from the vulva of N2 nematode colonized with Lp02 for 4 days. Note the vacuole containing motile coccoid-shaped bacteria (white arrow). Other vacuoles containing bacteria are present but on other planes out of focus. Scale bar is 2 *μ*m.

**Video S5.** Vacuoles containing GFP-expressed *Legionella pneumophila* in the gonadal tissue of *Caenorhabditis elegans* nematodes. Microscopic videos of a *glo-3*(*kx94*) nematode fed with Lp02 P_magA_::*gfp* strain for 8 days p.i. (A–C) represent DIC, green, and merge channels, respectively. Scale bar is 2 *μ*m. Note the multiple vacuoles containing motile coccoid-shaped bacteria. Also note that only some of the bacteria are fluorescent indicating that these bacterial forms are still metabolically active and in the final stages of developing into mature cyst forms. Fully developed cyst forms become metabolically dormant hence they lose the fluorescence due to the eventual degradation of GFP.

**Video S6.** *Caenorhabditis elegans* reproductive tract. DIC microscopy video of the reproductive tract of a young adult N2 nematode harvested from a NGM plate supplemented with *Escherichia coli* OP50. Note the anatomical features that include the U-shaped gonad (G) with an oocyte (O) located adjacent to the spermatheca (S) and an embryo (E) in the uterine region. Note that the sperm is distinct in size, shape and motility from the observed *Legionella pneumophila* rod- and coccoid-shaped bacteria in vacuoles in infected nematodes. Scale bar is 5 *μ*m.

## References

[b1] Aballay A, Ausubel FM (2001). Programmed cell death mediated by *ced-3* and *ced-4* protects *Caenorhabditis elegans* from *Salmonella typhimurium*-mediated killing. Proc. Natl. Acad. Sci. USA.

[b2] Aballay A, Yorgey P, Ausubel FM (2000). *Salmonella typhimuirum* proliferates and establishes a persistent infection in the intestine of *Caenorhabditis elegans*. Curr. Biol.

[b3] Abdelhady H, Garduño RA (2013). The progeny of *Legionella pneumophila* in human macrophages shows unique developmental traits. FEMS Microbiol. Lett.

[b4] Agbor TA, McCormick BA (2011). Salmonella effectors: important players modulating host cell function during infection. Cell. Microbiol.

[b5] Albertson DG, Thomson JN (1976). The pharynx of *Caenorhabditis elegans*. Philos. Trans. R. Soc. Lond. B Biol. Sci.

[b6] Albert-Weissenberger C, Cazalet C, Buchrieser C (2007). *Legionella pneumophila* – a human pathogen that co-evolved with fresh water protozoa. Cell. Mol. Life Sci.

[b7] Altun ZF, Hall DH (2009). 10.3908/wormatlas.1.4.

[b8] Anderson GL, Kenney SJ, Millner PD, Beuchat LR, Williams PL (2006). Shedding of foodborne pathogens by *Caenorhabditis elegans* in compost-amended and unamended soil. Food Microbiol.

[b9] Avery L (1993). The genetics of feeding in *Caenorhabditis elegans*. Genetics.

[b10] Avery L, Riddle DL, Blumenthal T, Meyer BJ, Preiss JR, Thomas JH (1997). Feeding and defecation. C. elegans II.

[b11] Balla KM, Troemel ER (2013). *Caenorhabditis elegans* as a model for intracellular pathogen infection. Cell. Microbiol.

[b12] Banga S, Gao P, Shen X, Fiscus V, Zong WX, Chen L (2007). *Legionella pneumophila* inhibits macrophage apoptosis by targeting pro-death members of the Bcl2 protein family. Proc. Natl. Acad. Sci. USA.

[b13] Berger KH, Isberg RR (1993). Two distinct defects in intracellular growth complemented by a single genetic locus in *Legionella pneumophila*. Mol. Microbiol.

[b14] Berger KH, Merriam JJ, Isberg RR (1994). Altered intracellular targeting properties associated with mutations in the *Legionella pneumophila dotA* gene. Mol. Microbiol.

[b15] Berk SG, Ting RS, Turner GW, Ashburn RJ (1998). Production of respirable vesicles containing live *Legionella pneumophila* cells by two *Acanthamoeba* spp. Appl. Environ. Microbiol.

[b16] Berk SG, Faulkner G, Garduño E, Joy MC, Ortiz-Jimenez MA, Garduño RA (2008). Packaging of live *Legionella pneumophila* into pellets expelled by *Tetrahymena* spp. does not required bacterial replication and depends on a Dot/Icm-mediated survival mechanism. Appl. Environ. Microbiol.

[b17] den Boer JW, Yzerman EP, Jansen R, Bruin JP, Verhoef LP, Neve G (2007). Legionnaires’ disease and gardening. Clin. Microbiol. Infect.

[b18] Brassinga AKC, Sifri CD (2013). The *Caenorhabditis elegans* model of *Legionella* infection. Methods Mol. Biol.

[b19] Brassinga AKC, Kinchen JM, Cupp ME, Day SR, Hoffman PS, Sifri CD (2010). *Caenorhabditis* is a metazoan host for *Legionella*. Cell. Microbiol.

[b1001] Brenner S (1974). The genetics of Caenorhabditis elegans. Genetics.

[b20] Brodsky FM, Chen CY, Knuehl C, Towler MC, Wakeham DE (2001). Biological basket weaving: formation and function of clathrin-coated vesicles. Annu. Rev. Cell Dev. Biol.

[b1002] Bryan A, Abbott ZD, Swanson MS (2013). Constructing unmarked gene deletions in Legionella pneumophila. Methods Mol Biol.

[b21] Byrne B, Swanson MS (1998). Expression of *Legionella pneumophila* virulence traits in response to growth conditions. Infect. Immun.

[b22] Casati S, Gioria-Martinoni A, Gaia V (2009). Commercial potting soils as an alternative infection source of *Legionella pneumophila* and other *Legionella* species in Switzerland. Clin. Microbiol. Infect.

[b23] Chong A, Lima CA, Allan DS, Nasrallah GK, Garduño RA (2009). The purified and recombinant chaperonin alters mitochondrial trafficking and microfilament organization. Infect. Immun.

[b24] Cirillo JD, Falkow S, Tompkins LS (1994). Growth of *Legionella pneumophila* in *Acanthamoeba castellanii* enhances invasion. Infect. Immun.

[b25] Cirillo JD, Cirillo SL, Yan L, Bermudez LE, Falkow S, Tompkins LS (1999). Intracellular growth in *Acanthamoeba castellanii* affects monocyte entry mechanisms and enhances virulence of *Legionella pneumophila*. Infect. Immun.

[b26] Clark L, Martinez-Argundo I, Humphrey TJ, Jepson MA (2009). GFP plasmid-induced defects in *Salmonella* invasion depend on plasmid architecture, not protein expression. Microbiology.

[b27] Clokey GV, Jacobsen LA (1986). The autofluorescent “lipofuscin granules” in the intestinal cells of *Caenorhabditis elegans* are secondary lysosomes. Mech. Ageing Dev.

[b28] Collins JJ, Huang C, Hughes S, Kornfeld K, WormBook (2007). The measurement and analysis of age-related changes in Caenorhabditis elegans.

[b29] Conradt B, Horvitz HR (1999). The TRA-1A sex determination protein of *C. elegans* regulates sexually dimorphic cell deaths by repressing the *egl-1* cell death activator gene. Cell.

[b30] Creasey EA, Isberg RR (2012). The protein SdhA maintains the integrity of the *Legionella*-containing vacuole. Proc. Natl. Acad. Sci. USA.

[b31] Dorer MS, Kirton D, Bader JS, Isberg RR (2006). RNA interference analysis of *Legionella* in *Drosophila* cells: exploitation of early secretory apparatus dynamics. PLoS Pathog.

[b32] Edwards RL, Dalebroux ZD, Swanson MS (2009). *Legionella pneumophila* couples fatty acid flux to microbial differentiation and virulence. Mol. Microbiol.

[b1000] Escoll P, Rolando M, Gomez-Valero L, Buchreiser C (2013). From amoeba to macrophages: exploring the molecular mechanisms of Legionella pneumophila infection in both hosts. Curr Top Microbiol Immunol.

[b33] Estes KA, Szumowski SC, Troemel ER (2011). Non-lytic, actin-based exit of intracellular parasites from *C. elegans* intestinal cells. PLoS Pathog.

[b34] Fang-Yen C, Avery L, Samuel ADT (2009). Two size-selective mechanisms specifically trap bacteria-sized food particles in *Caenorhabditis elegans*. Proc. Natl. Acad. Sci. USA.

[b35] Fares H, Grant B (2002). Deciphering endocytosis in *Caenorhabditis elegans*. Traffic.

[b36] Faulkner G, Garduño RA (2002). Ultrastructural analysis of differentiation in *Legionella pneumophila*. J. Bacteriol.

[b37] Faulkner G, Garduño RA (2013). Electron microscopy of *Legionella* and *Legionella*-infected cells. Methods Mol. Biol.

[b38] Feeley JC, Gibson RJ, Gorman GW, Langford NC, Rasheed JK, Mackel DC, Baine WB (1979). Charcoal-yeast extract agar: primary isolation medium for Legionella pneumophila. J Clin Microbiolol.

[b39] Fields BS (1996). The molecular ecology of legionellae. Trends Microbiol.

[b40] Fields BS, Benson RF, Besser RE (2002). Legionella and Legionnaires’ disease: 25 years of investigation. Clin. Microbiol. Rev.

[b41] Fonseca MV, Swanson MS (2014). Nutrient salvaging and metabolism by the intracellular pathogen *Legionella pneumophila*. Front. Cell. Infect. Microbiol.

[b42] Garduño RA, Heuner K, Swanson MS (2008). Developmental cycle – differentiation of *Legionella pneumophila*. Legionella molecular microbiology.

[b43] Garduño RA, Garduño E, Hoffman PS (1998). Surface-associated hsp60 chaperonin of *Legionella pneumophila* mediates invasion in a HeLa cell model. Infect. Immun.

[b44] Garduño RA, Garduño E, Hiltz M, Hoffman PS (2002). Intracellular growth of *Legionella pneumophila* gives rise to a differentiated form dissimilar to stationary-phase forms. Infect. Immun.

[b45] Gartner A, Boag PR, Blackwell TK, WormBook (2008). Germline survival and apoptosis.

[b46] Gibbs DS, Anderson DL, Beuchat LR, Carta LK, Williams PL (2005). Potential role of *Diploscapter* sp. strain LKC25, a bacterivorous nematode from soil, as a vector of food-borne pathogenic bacteria to preharvest fruits and vegetables. Appl. Environ. Microbiol.

[b47] Grant B, Hirsh D (1999). Receptor-mediated endocytosis in the *Caenorhabditis elegans* oocyte. Mol. Biol. Cell.

[b48] Grant BD, Sato M (2006). http://www.wormbook.org.

[b49] Grant B, Zhang Y, Paupard MC, Lin SX, Hall DH, Hirsh D (2001). Evidence that RME-1, a conserved *C. elegans* EH-domain protein, functions in endocytic recycling. Nat. Cell Biol.

[b50] Greub G, Raoult D (2003). Morphology of *Legionella pneumophila* according to their location within *Hartmanella vermiformis*. Res. Microbiol.

[b51] Grewal PS, Wright DJ (1992). Migration of *Caenorhabditis elegans* larvae towards bacteria and the nature of the bacterial stimulus. Fundam. Appl. Nematol.

[b52] Hägele S, Köhler R, Merkert H, Schleicher M, Hacker J, Steinert M (2000). *Dictyostelium discoideum*: a new host model system for intracellular pathogens of the genus *Legionella*. Cell. Microbiol.

[b53] Hall DH (1995). Electron microscopy and three-dimensional image reconstruction. Methods Cell Biol.

[b54] Hammer BK, Swanson MS (1999). Co-ordination of *Legionella pneumophila* virulence into stationary phase by ppGpp. Mol. Microbiol.

[b55] Hedgecock EM, Sulston JE, Thomson JN (1983). Mutations affecting programmed cell deaths in the nematode *Caenorhabditis elegans*. Science.

[b56] Hiltz MF, Sisson GR, Brassinga AK, Garduño E, Garduño RA, Hoffman PS (2004). Expression of *magA* in *Legionella pneumophila* Philadelphia-1 is developmentally regulated and a marker of formation of mature intracellular forms. J. Bacteriol.

[b57] Hodgkin J, Kuwabara PE, Corneliussen B (2000). A novel bacterial pathogen, *Microbacterium nematophilum*, induces morphological change in the nematode *C. elegans*. Curr. Biol.

[b58] Hope IA, Hope IA (1999). *C. elegans*: a practical approach. The practical approach series.

[b59] Hovel-Miner G, Faucher SP, Charpentier X, Shuman HA (2010). ArgR-regulated genes are derepressed in the *Legionella*-containing vacuole. J. Bacteriol.

[b60] Hubber A, Roy CR (2010). Modulation of host cell function by *Legionella pneumophila* type IV effectors. Annu. Rev. Cell Dev. Biol.

[b61] Hughes MS, Steele TW (1994). Occurrence and distribution of *Legionella* species in composted plant materials. Appl. Environ. Microbiol.

[b62] Irazoqui JE, Troemel ER, Feinbaun RL, Luhachack LG, Cezairliyan BO, Ausubel FM (2010). Distinct pathogenesis and host responses during infection of *C. elegans* by *P. aeruginosa* and *S. aureus*. PLoS Pathog.

[b63] Isberg RR, O’Conner TJ, Heidtman M (2009). The *Legionella pneumophila* replication vacuole: making a cosy niche inside host cells. Nat. Rev. Microbiol.

[b64] Jia K, Thoman C, Akbar M, Sun Q, Adams-Huet B, Gilpin C (2009). Autophagy genes protect against *Salmonella typhimurium* infection and mediate insulin signaling-regulated pathogen resistance. Proc. Natl. Acad. Sci. USA.

[b65] Kimble J, Sharrock WJ (1983). Tissue-specific synthesis of yolk proteins in *Caenorhabditis elegans*. Dev. Biol.

[b66] Kinchen JM, Hengartner MO (2005). Tales of cannibalism, suicide, and murder: programmed cell death in *C. elegans*. Curr. Top. Dev. Biol.

[b67] Knodler LA, Bestor A, Ma C, Hansen-Wester I, Hensel M, Vallance BA (2005). Cloning vectors and fluorescent proteins can significantly inhibit *Salmonella enterica* virulence in both epithelial cells and macrophages: implications for bacterial pathogenesis studies. Infect. Immun.

[b68] Laguna RK, Creasey EA, Li Z, Valtz N, Isberg RR (2006). A *Legionella pneumophila*-translocated substrate that is required for growth within macrophages and protection from host cell death. Proc. Natl. Acad. Sci. USA.

[b69] Lau HY, Ashbolt NJ (2009). The role of biofilms and protozoa in *Legionella* pathogenesis: implications for drinking water. J. Appl. Microbiol.

[b70] Lehane MJ (1997). Peritrophic matrix structure and function. Annu. Rev. Entomol.

[b71] Matyash V, Geier C, Henske A, Mukherjee S, Hirsh D, Thiele C (2001). Distribution and transport of cholesterol in *Caenorhabditis elegans*. Mol. Biol. Cell.

[b72] McGhee J, WormBook (2007). The C. elegans intestine.

[b73] Merriam JJ, Mathur R, Maxfield-Boumil R, Isberg RR (1997). Analysis of the *Leigonella pneumophila flil* gene: intracellular growth of a defined mutant defective for flagellum biosynthesis. Infect. Immun.

[b74] Moffat JF, Tompkins LS (1992). A quantitative model of intracellular growth of *Legionella pneumophila* in *Acanthamoena castellanii*. Infect. Immun.

[b75] Molofsky AB, Swanson MS (2004). Differentiate to thrive: lessons from the *Legionella pneumophila* life cycle. Mol. Microbiol.

[b76] Morash MG, Brassinga AKC, Warthan M, Gourabathini P, Garduño RA, Goodman SD (2009). Reciprocal expression of integration host factor and HU in the developmental cycle and infectivity of *Legionella pneumophila*. Appl. Environ. Microbiol.

[b77] Newton HJ, Ang DKY, van Driel IR, Hartland EL (2010). Molecular pathogenesis of infections caused by *Legionella pneumophila*. Clin. Microbiol. Rev.

[b78] Nicholas HR, Hodgkin J (2004). The ERK MAP kinase cascade mediates tail swelling and protective response to rectal infection in *C. elegans*. Curr. Biol.

[b79] Rabbitts BM, Ciotti MK, Miller NE, Kramer M, Lawrenson AL, Levitte S (2008). *glo-3*, a novel *Caenorhabditis elegans* gene, is required for lysosome-related organelle biogenesis. Genetics.

[b80] Robertson P, Abdelhady H, Garduño RA (2014). The many forms of a pleomorphic bacterial pathogen – the developmental network of *Legionella pneumophila*. Front. Microbiol.

[b81] Rodger S, Griffiths BS, McNicol JW, Wheatley RW, Young IM (2004). The impact of bacterial diet on the migration and navigation of *Caenorhabditis elegans*. Microb. Ecol.

[b82] Roy CR, Berger KH, Isberg RR (1998). *Legionella pneumophila* DotA protein is required for early phagosome trafficking decisions that occur within minutes of bacterial uptake. Mol. Microbiol.

[b83] Sambrook J (2001). Molecular cloning: a laboratory manual/Joseph Sambrook, David W. Russell.

[b84] Sato K, Norris A, Sato M, Grant BD (2014). C. elegans as a model for membrane traffic (April 25, 2014).

[b85] Schneider WJ (1996). Vitellogenin receptors: oocyte-specific members of the low-density lipoprotein receptor supergene family. Int. Rev. Cytol.

[b86] Schumacher B, Schertel C, Wittenburg N, Tuck S, Mitani S, Gartner A (2005). *C. elegans ced-13* can promote apoptosis and is induced in response to DNA damage. Cell Death Differ.

[b87] Sharrock WJ, Sutherlin ME, Leske K, Cheng TK, Kim TY (1990). Two distinct yolk lipoprotein complexes form *Caenorhabditis elegans*. J. Biol. Chem.

[b88] Sifri CD, Begun J, Ausubel FM (2005). The worm has turned – microbial virulence modeled in *Caenorhabditis elegans*. Trends Microbiol.

[b89] Srikanth CV, Wall DM, Maldonado-Contreras A, Shi HN, Zhou D, Demma Z (2010). Salmonella pathogenesis and processing of secreted effectors by caspase-3. Science.

[b90] Tenor JL, Aballay A (2008). A conserved Toll-like receptor is required for Caenorhabditis elegans innate immunity. EMBO Rep.

[b91] Timmons L, Court DL, Fire A (2001). Ingestion of bacterially expressed dsRNAs can produce specific and potent genetic interference in *Caenorhabditis elegans*. Gene.

[b92] Troemel ER, Félix MA, Whiteman NK, Barrière A, Ausubel FM (2008). Microsporidia are natural intercellular parasites of the nematode *Caenorhabditis elegans*. PLoS Biol.

[b93] Whiley H, Taylor M, Bentham R (2011). Detection of *Legionella* species in potting mixes using fluorescent in situ hybridization (FISH). J. Microbiol. Methods.

[b94] Young IM, Griffiths BS, Robertson WM (1996). Continuous foraging by bacterial-feeding nematodes. Nematologica.

[b95] Young IM, Griffiths BS, Robertson WM, McNicol JW (1998). Nematode (*Caenorhabditis elegans*) movement in sand as affected by particle size, moisture and the presence of bacteria (*Escherichia coli*. Eur. J. Soil Sci.

[b96] Zhang Y, Grant B, Hirsh D (2001). RME-8, a conserved J-domain protein, is required for endocytosis in *Caenorhabditis elegans*. Mol. Biol. Cell.

