# Supplementary figures and images for: Identification of vacuoles containing extraintestinal differentiated forms of Legionella pneumophila in colonized Caenorhabditis elegans soil nematodes
Source: Microbiologyopen. 2015 Jul 1;4(4):660–81. doi: 10.1002/mbo3.271 (PMC4554460; doi:10.1002/mbo3.271)

Supplemental Figure 1

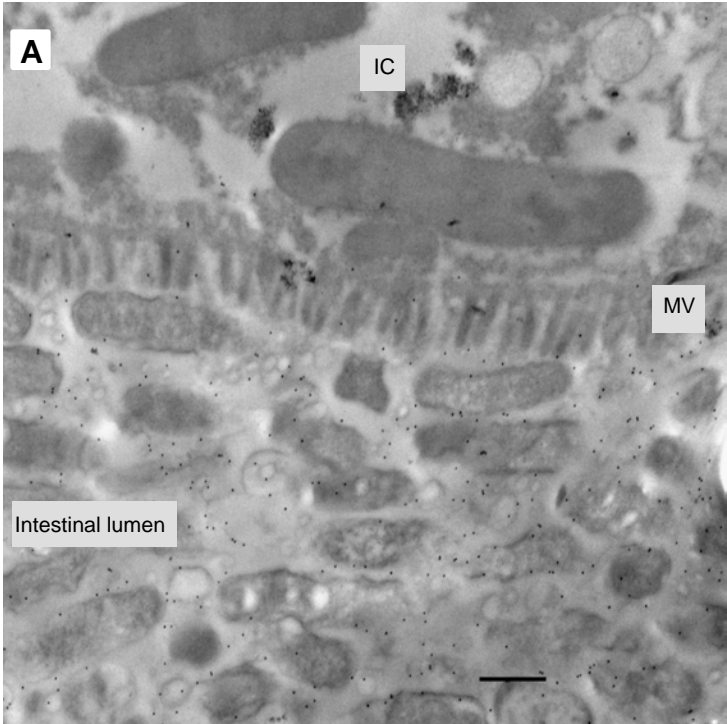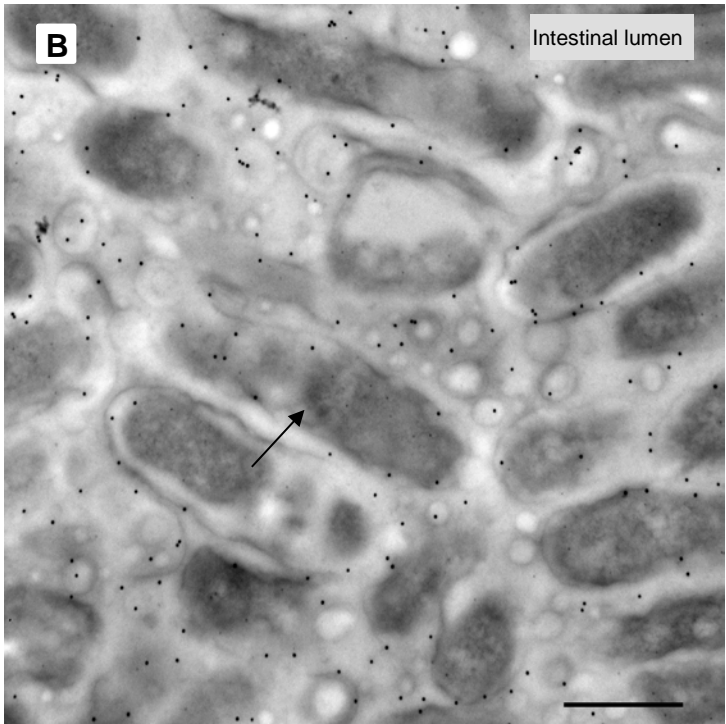

Supplemental Figure 2

**A**

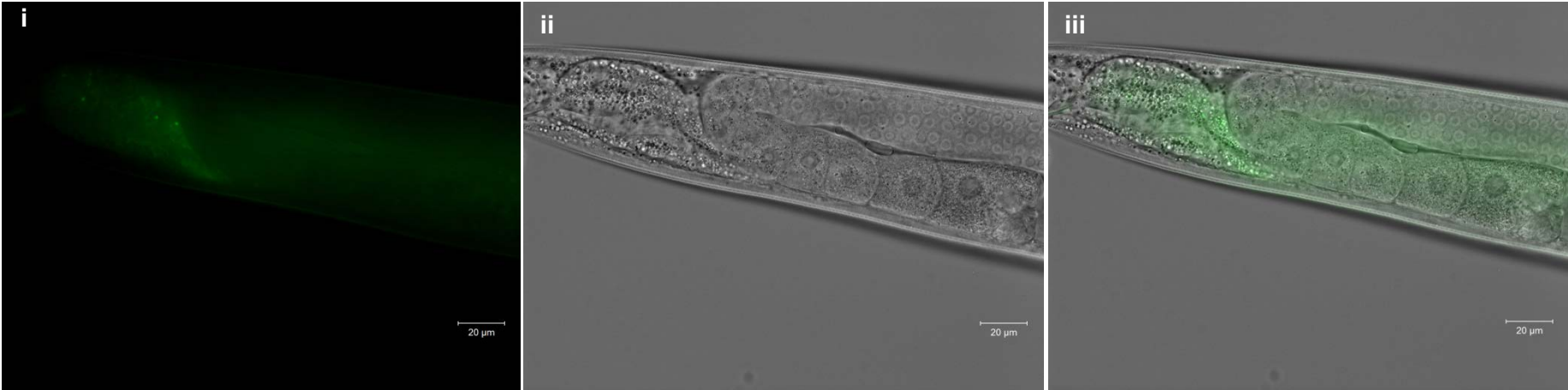

**B**

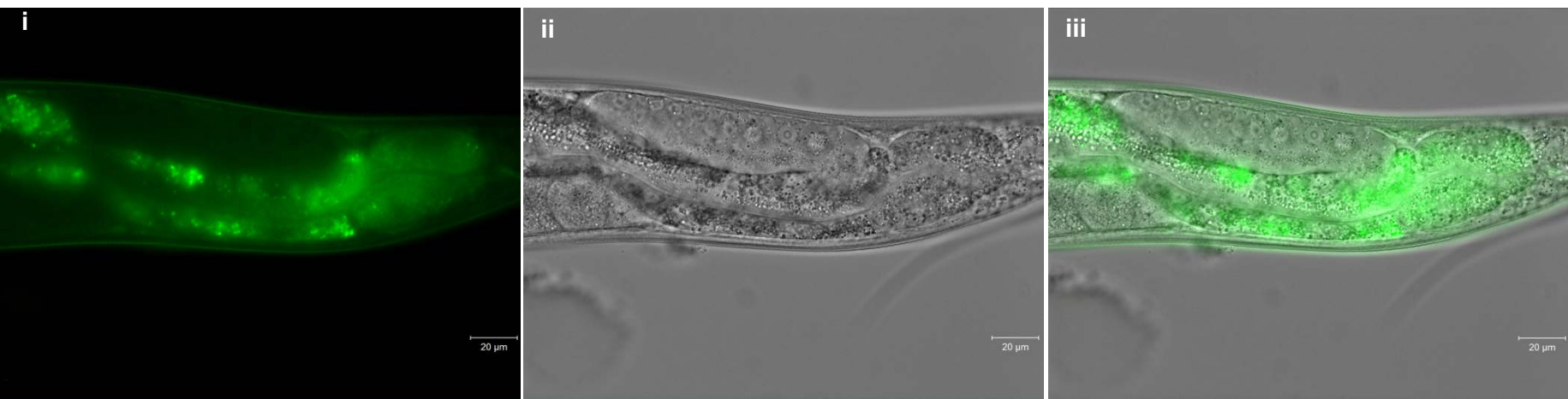

Supplement: Supplementary file 1 [file mbo30004-0660-sd1.pdf]
